# Guided co-clustering transfer across unpaired and paired single-cell multi-omics data

**DOI:** 10.1093/bioinformatics/btaf639

**Published:** 2025-12-01

**Authors:** Hongyao Li, Yunrui Liu, Pengcheng Zeng

**Affiliations:** Institute of Mathematical Sciences, ShanghaiTech University, Shanghai, 201210, China; Institute of Mathematical Sciences, ShanghaiTech University, Shanghai, 201210, China; Institute of Mathematical Sciences, ShanghaiTech University, Shanghai, 201210, China

## Abstract

**Motivation:**

Single-cell multi-omics technologies enable the simultaneous profiling of gene expression and chromatin accessibility, providing complementary insights into cellular identity and gene regulatory mechanisms. However, integrating paired scRNA-seq and scATAC-seq data (i.e. profiles from the same single cell) remains challenging due to inherent sparsity, technical noise, and the limited availability of high-quality paired measurements. In contrast, large-scale unpaired scRNA-seq datasets often exhibit robust and biologically meaningful cell cluster structures.

**Results:**

We introduce *Guided Co-clustering Transfer (GuidedCoC)*, a novel framework that transfers structural knowledge from unpaired scRNA-seq source data to improve both cell clustering and feature alignment in paired scRNA-seq/scATAC-seq target data. GuidedCoC jointly co-cluster cells and features across modalities and domains via a unified information-theoretic objective, aligning gene expression modules with regulatory elements while implicitly performing cross-modal dimensionality reduction to reduce noise. Additionally, it automatically aligns cell populations across unpaired and paired datasets without requiring explicit annotations. Extensive experiments on multiple benchmark datasets demonstrate that GuidedCoC achieves superior clustering accuracy and biological interpretability compared to existing methods. These results highlight the promise of structure-guided transfer learning for robust, scalable, and interpretable integration of single-cell multi-omics data.

**Availability and implementation:**

GuidedCoC is available as open-source code at https://github.com/No-AgCl/GuidedCoC.

## 1 Introduction

Recent advances in single-cell multi-omics technologies have revolutionized our ability to dissect cellular heterogeneity by simultaneously profiling gene expression (scRNA-seq) and chromatin accessibility (scATAC-seq) at single-cell resolution from the same cell (Macaulay *et al.* 2017). These paired modalities offer complementary insights: scRNA-seq captures transcriptional states, while scATAC-seq identifies regulatory elements governing cell identity and function (Stuart *et al.* 2019, Chen *et al.* 2019). However, joint analysis of these data remains challenging due to technical noise, sparsity, and batch effects, particularly in emerging datasets where paired measurements are often limited in quality or scale (Argelaguet *et al.* 2018). Concurrently, large-scale single-cell atlases, such as Tabula Muris and Human Cell Atlas, provide high-quality unpaired scRNA-seq data with inherent cluster structures that approximate known cell types (Regev *et al.* 2017, Tabula Muris Consortium 2020). This presents an opportunity to leverage such biologically meaningful structures from the reference data (i.e. unpaired scRNA-seq data) to enhance analysis of sparse, noisy multi-omics data (i.e. paired scRNA-seq/scATAC-seq data)—a paradigm shift from traditional single-dataset clustering.

Computational integration methods have evolved rapidly but face critical limitations. Early efforts in integrating paired scRNA-seq and scATAC-seq data primarily focused on latent space alignment across modalities. Methods such as MOFA+ (Argelaguet *et al.* 2020) and Seurat (Hao *et al.* 2021, 2024) apply dimensionality reduction to jointly embed multi-modal data, facilitating downstream clustering and visualization, but they often neglect feature-level alignment and modality-specific signal. More expressive models, such as Cobolt (Gong *et al.* 2021), leverage hierarchical variational autoencoders to disentangle shared and private representations, while BABEL (Wu *et al.* 2021) performs cross-modal prediction to impute missing modalities. MultiVI (Ashuach *et al.* 2023) further extends probabilistic modeling to integrate and augment sparse, paired multi-omics profiles. However, these methods largely rely on fully paired data and can struggle under extreme sparsity (Lance *et al.* 2022). To move beyond full pairing, scMoMaT (Zhang *et al.* 2023) addresses mosaic integration through matrix tri-factorization, aligning cells and features across batches and modalities. While effective, these methods often overlook the potential of external, unpaired datasets to scaffold clustering in target data. Furthermore, they struggle with feature-level alignment, such as linking gene modules in scRNA-seq to regulatory peaks in scATAC-seq, which is critical for interpreting transcriptional regulation (Pliner *et al.* 2018). A recent benchmarking study (Lee *et al.* 2023) assesses many of these methods.

Transfer learning has emerged as a powerful strategy for integrative analysis of single-cell data, yet most approaches [e.g. scArches (Lotfollahi *et al.* 2022), singleCellNet (Tan and Cahan 2019)] rely on explicit cell-type labels from reference datasets, limiting their applicability to unannotated or novel cell states. The co-clustering-based transfer learning methods, *couple*CoC (Zeng *et al.* 2021) and *couple*CoC+ (Zeng and Lin 2021), can freely leverage cell-type labels from source datasets. However, neither method is suitable for integrating paired scRNA-seq and scATAC-seq data. Unsupervised methods like SCOT (Demetci *et al.* 2022) and SMAI (Ma *et al.* 2024) align datasets via optimal transport or manifold learning but focus solely on cell-level correspondences, ignoring cross-modal feature relationships. This is particularly problematic for multi-omics integration, where regulatory logic (e.g. transcription factor binding sites modulating gene expression) must be preserved (Pliner *et al.* 2018). Biological evidence supports the transferability of latent structures: studies like the Tabula Muris (Tabula Muris Consortium 2020) and cross-species analyses by Haghverdi *et al.* (2018) show that gene modules (e.g. lineage-specific programs) are often conserved across conditions. However, as highlighted in reviews by Lähnemann *et al.* (2020), computational tools have yet to fully exploit this property for unsupervised multi-omics guidance.

To address the aforementioned challenges, we propose *Guided Co-clustering Transfer (GuidedCoC)*, a novel information-theoretic co-clustering framework that integrates paired scRNA-seq/scATAC-seq data with unpaired scRNA-seq data. GuidedCoC is biologically motivated by two key observations: (i) high-quality unpaired scRNA-seq datasets, even without explicit labels, encode robust cluster structures reflective of cell identity (Tabula Muris Consortium 2020); and (ii) gene activity scores or promoter accessibility derived from paired scATAC-seq data serve as a natural bridge to paired scRNA-seq features, enabling cross-modal feature co-clustering (Schep *et al.* 2017). By formulating a joint optimization over cell and feature clusters, GuidedCoC transfers knowledge from unpaired scRNA-seq data to stabilize clustering in paired scRNA-seq/scATAC-seq data, while aligning gene–peak modules to enhance biological interpretability.

This work makes the following key contributions: (i) *New Problem Setting*. We investigate a realistic yet underexplored scenario—knowledge transfer across unpaired and paired single-cell multi-omics data—by leveraging the inherent structure in unlabeled, unpaired scRNA-seq source data to guide clustering in paired scRNA-seq/scATAC-seq target data. (ii) Novel Methodology. GuidedCoC builds upon and extends classical information-theoretic co-clustering (Dhillon *et al.* 2003), enabling simultaneous clustering of cells and co-clustering of gene expression and chromatin accessibility features. This cross-modal feature co-clustering performs implicit dimension reduction in a shared feature space, mitigating noise and sparsity while aligning transcriptional modules with regulatory elements. The framework also facilitates automatic alignment of similar cell populations across unpaired and paired datasets, bridging the gap between single-modality transfer learning and multi-omics integration. (iii) Comprehensive Evaluation. Extensive experiments on multiple benchmark datasets demonstrate the effectiveness of GuidedCoC over state-of-the-art methods in terms of clustering accuracy and biological interpretability, highlighting its potential for scalable and robust analysis of sparse and noisy single-cell multi-omics data.

The remainder of this paper is structured as follows. In Section 2, we introduce our methodological framework, beginning with a systematic analysis of existing co-clustering approaches across diverse datasets. We then detail the proposed guided co-clustering transfer learning model, along with the associated feature selection strategies, data preprocessing procedures, and evaluation metrics. Section 3 presents comprehensive experimental results, benchmarking our method against state-of-the-art baselines. Finally, Section 5 summarizes the key findings and highlights potential directions for future research.

## 2 The methodology

**Figure 1.**
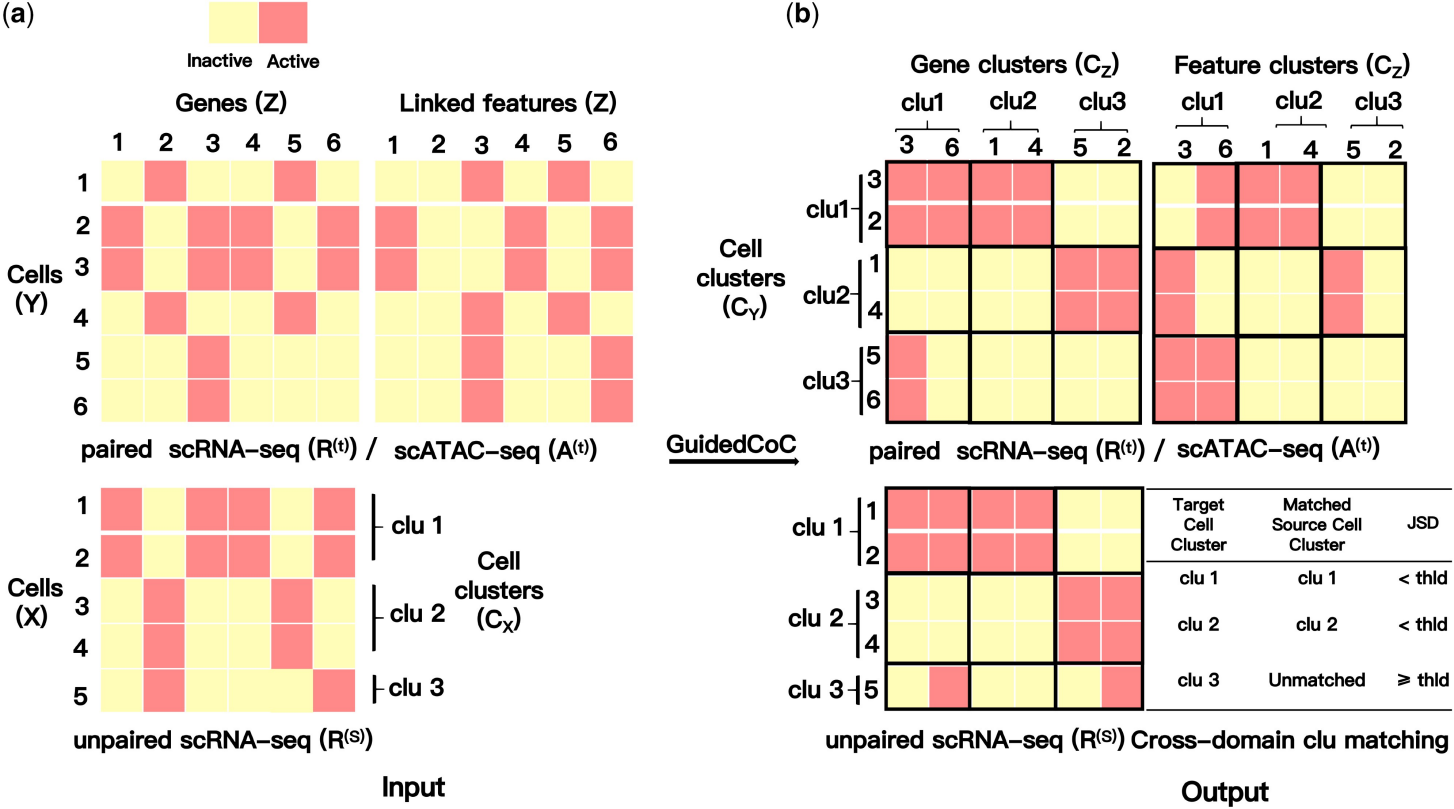
A toy example of *GuidedCoC*. (a) The input includes paired target datasets $R^{\left(t\right)}$ and $A^{\left(t\right)}$, where gene activity scores (or promoter accessibility) in $A^{\left(t\right)}$ are linked to genes in $R^{\left(t\right)}$, and a source scRNA-seq dataset $R^{\left(s\right)}$ that shares the same gene set and has known cell clusters $C_{X}$. (b) The output includes shared feature clusters $C_{Z}$, target cell clusters $C_{Y}$, and cross-domain cluster matching for shared cell populations between the source and target data. “clu”: cluster; “thld”: threshold.

We address a structure-guided transfer clustering problem involving three datasets: (i) paired scRNA-seq data $R^{\left(t\right)}$ and scATAC-seq data $A^{\left(t\right)}$, which constitute the *target* and are typically noisy and sparse; and (ii) unpaired scRNA-seq data $R^{\left(s\right)}$, serving as the *source*, assumed to be of higher quality with robust cluster structure but without explicit labels (Fig. 1a). We assume that a subset of features in $A^{\left(t\right)}$—specifically gene activity score or promoter accessibility—are linked to $R^{\left(t\right)}$ and share clustering structure with the source scRNA-seq data $R^{\left(s\right)}$. This enables potential alignment at both the cell and feature levels. Our goal is to transfer structural knowledge—cell clusters and feature modules—from the source data $R^{\left(s\right)}$ to improve clustering and feature alignment in the target multi-omics data $R^{\left(t\right)}$/$A^{\left(t\right)}$, and also to enable cross-domain cluster matching for shared cell populations between the source and target data (Fig. 1b).

### 2.1 Co-clustering for each dataset

We begin by considering the target domain’s paired dataset $R^{\left(t\right)}$, represented as an $n^{\left(t\right)}\times q$ matrix, containing *q* features measured across $n^{\left(t\right)}$ cells. Let *Y* and *Z* denote discrete random variables corresponding to cell and feature indices, respectively, where $Y\in \{1,\ldots ,n^{\left(t\right)}\}$ and Z∈{1,…,q}. The joint probability $P_{R^{\left(t\right)}}(Y,Z)$ captures the activity of feature *z* in cell *y*, estimated by normalizing the data matrix so that the total sum equals one:


(1) $$P_{R^{\left(t\right)}}(Y=y,Z=z)=\frac{{\left(R^{\left(t\right)}\right)}_{yz}}{\sum_{y=1}^{n^{\left(t\right)}}\sum_{z=1}^{q}{\left(R^{\left(t\right)}\right)}_{yz}}.$$

The goal of co-clustering is to group similar cells and features. Assume $N^{\left(t\right)}$ cell clusters and *K* feature clusters. Define $\tilde{Y}\in \{1,\ldots ,N^{\left(t\right)}\}$ and $\tilde{Z}\in \{1,\ldots ,K\}$ as the corresponding cluster variables, determined by clustering functions $C_{Y}$ and $C_{Z}$, such that $C_{Y}(y)=\tilde{y}$ and $C_{Z}(z)=\tilde{z}$. The joint distribution over clusters is then:


(2) $${\tilde{P}}_{R^{\left(t\right)}}(\tilde{Y}=\tilde{y},\tilde{Z}=\tilde{z})=\sum_{\left\{y:C_{Y}(y)=\tilde{y}\right\}}\sum_{\left\{z:C_{Z}(z)=\tilde{z}\right\}}P_{R^{\left(t\right)}}(y,z).$$

We now define a clustering loss grounded in the KL divergence between the joint cell-feature distribution and its constructed reference:


(3) $$L_{R^{\left(t\right)}}(C_{Y},C_{Z})=D_{\text{KL}}\left(P_{R^{\left(t\right)}}(Y,Z)\|P_{R^{\left(t\right)}}^{*}(Y,Z)\right),$$

where the reference $P_{R^{\left(t\right)}}^{*}$ adjusts a coarse-grained distribution using marginals to approximate cluster-based independence, and $P_{R^{\left(t\right)}}^{*}$ is structured as:


(4) $$P_{R^{\left(t\right)}}^{*}(Y=y,Z=z)={\tilde{P}}_{R^{\left(t\right)}}(\tilde{Y},\tilde{Z})\cdot \frac{P_{R^{\left(t\right)}}(Y=y)}{{\tilde{P}}_{R^{\left(t\right)}}(\tilde{Y})}\cdot \frac{P_{R^{\left(t\right)}}(Z=z)}{{\tilde{P}}_{R^{\left(t\right)}}(\tilde{Z})},$$

where $\tilde{Y}=C_{Y}(y)$ and $\tilde{Z}=C_{Z}(z)$ are the cluster indices. The objective $L_{R^{\left(t\right)}}$ in Equation (3) equivalently minimizes the loss of mutual information between cells and features during co-clustering, as established in the information-theoretic co-clustering framework (Dhillon *et al.* 2003, Dai *et al.* 2008).

For the auxiliary target modality $A^{\left(t\right)}$, represented as an $n^{\left(t\right)}\times q$ matrix, sharing cell and feature identities with $R^{\left(t\right)}$ (features from both datasets are profiled from the same single cell and are linked), we reuse *Y*, $C_{Y}$, *Z* and $C_{Z}$:


(5) $$L_{A^{\left(t\right)}}(C_{Y},C_{Z})=D_{\text{KL}}\left(P_{A^{\left(t\right)}}(Y,Z)||P_{A^{\left(t\right)}}^{*}(Y,Z)\right).$$

For the source data $R^{\left(s\right)}$ (where the cell clustering function $C_{X}$ is known with $|C_{X}|=N^{\left(s\right)}$ clusters, and feature identities are shared across domains), represented as an $n^{\left(s\right)}\times q$ matrix, we reuse *Z* and $C_{Z}$:


(6) $$L_{R^{\left(s\right)}}(C_{Z}|C_{X})=D_{\text{KL}}\left(P_{R^{\left(s\right)}}(X,Z)||P_{R^{\left(s\right)}}^{*}(X,Z)\right).$$

$P_{A^{\left(t\right)}}^{*}$ and $P_{R^{\left(s\right)}}^{*}$ mirror $P_{R^{\left(t\right)}}^{*}$’s structure [Equation (4)].

### 2.2 Guided co-clustering transfer learning

We integrate paired/unpaired single-cell multi-modal data via joint clustering of cells and features across domains. The framework has two stages: (i) integrative co-clustering of $R^{\left(t\right)}$, $A^{\left(t\right)}$, and $R^{\left(s\right)}$, and (ii) cross-domain matching of shared cell populations.

#### 2.2.1 Stage 1: Integrative co-clustering

The final co-clustering objective jointly minimizes loss across modalities and domains:


(7) $$\underset{C_{Y},C_{Z}}{\min}\;\underset{\text{scRNA-target}}{\underset{︸}{L_{R^{\left(t\right)}}(C_{Y},C_{Z})}}+\alpha \underset{\text{scATAC-target}}{\underset{︸}{L_{A^{\left(t\right)}}(C_{Y},C_{Z})}}+\beta \underset{\text{scRNA-source}}{\underset{︸}{L_{R^{\left(s\right)}}(C_{Z}|C_{X})}}$$

with hyperparameters α,β controlling modality and domain contributions. The key idea is that the clustering function $C_{Z}$ over features acts as a conduit for transferring knowledge from the source data to the target domain. Given high-quality clustering $C_{X}$ in $R^{\left(s\right)}$, the learned feature clusters $C_{Z}$ improve both integration and robustness across domains. Furthermore, by coupling $R^{\left(t\right)}$ and $A^{\left(t\right)}$ via shared clustering functions, we leverage modality-specific structure to enhance biological interpretability. Here, we empirically set the number of shared feature clusters to K=12, and the weighting hyperparameters to α=0.8 and β=1. For a detailed sensitivity analysis, please refer to Fig. 4 in Section 3.3.

The KL divergence for $R^{\left(t\right)}$ [Equation (3)] admits two symmetric decompositions—conditioning on cells or features—highlighting local consistency of cluster assignments (Dai *et al.* 2008, Zeng and Lin 2021):


(8) $$\begin{array}{cc} D_{\text{KL}}\left(P_{R^{\left(t\right)}}(Y,Z)\;||P_{R^{\left(t\right)}}^{*}(Y,Z)\right) & \\ =\sum_{i}\sum_{y\in C_{Y}^{-1}(i)}P_{R^{\left(t\right)}}(Y=y)D_{\text{KL}}(P_{R^{\left(t\right)}}(Z|Y=y)||P_{R^{\left(t\right)}}^{*}(Z|\tilde{Y}=i,Y=y)) & \\ =\sum_{j}\sum_{z\in C_{Z}^{-1}(j)}P_{R^{\left(t\right)}}(Z=z)D_{\text{KL}}(P_{R^{\left(t\right)}}(Y|Z=z)||P_{R^{\left(t\right)}}^{*}(Y|\tilde{Z}=j,Z=z)). & \end{array}$$

The KL divergences for $A^{\left(t\right)}$ [Equation (5)] and $R^{\left(s\right)}$ [Equation (6)] also mirror this structure. We optimize Equation (7) via block-coordinate descent:

- Updating $C_{Y}$ (cell clusters): Assign each *y* to cluster *i* via minimizing the weighted sum of KL divergences between conditional feature distributions in $R^{\left(t\right)}$ and $A^{\left(t\right)}$[Equation (A2), available as supplementary data at *Bioinformatics* online]
- Updating $C_{Z}$ (feature clusters): Assign each *z* to cluster *j* via minimizing a sum of KL divergences across all datasets: $R^{\left(t\right)}$, $A^{\left(t\right)}$, and $R^{\left(s\right)}$ [Equation (A3), available as supplementary data at *Bioinformatics* online]

The $C_{Y}$ update ensures cell-cluster consistency across modalities; the $C_{Z}$ update identifies functionally coherent features preserved across domains. Iterative refinement yields a unified latent representation, converging within 10 iterations (Fig. 5). Details (derivations, algorithm, complexity, convergence) are in Section A, available as supplementary data at *Bioinformatics* online.

#### 2.2.2 Stage 2: Cross-domain cluster matching

Given learned target domain clusters $C_{Y}$ and known source clusters $C_{X}$, the goal of the second stage is to align clusters that represent shared biological cell populations. We construct cluster-level joint distributions over features, denoted ${\tilde{p}}_{i}$ for source and ${\tilde{q}}_{j}$ for target clusters. Matching is performed by minimizing the average Jensen-Shannon divergence (AJSD) across $n_{\text{trials}}$ random subsamples:


(9) $$\mathrm{AJSD}({\tilde{p}}_{i},{\tilde{q}}_{j})=\frac{1}{n_{\text{trials}}}\sum_{l=1}^{n_{\text{trials}}}{\text{JSD}}^{\left(l\right)}({\tilde{p}}_{i},{\tilde{q}}_{j}).$$

Only cluster pairs with AJSD below a threshold $\tau_{\text{JSD}}$ are retained as valid matches. To avoid assignment conflicts and ensure optimal alignment, we repeat the matching over $n_{\text{shuffles}}$ permutations of $C_{Y}$, selecting the configuration $M^{*}$ that minimizes the summation of AJSD over all valid matches:


(10) $$M^{*}=\underset{M^{\left(r\right)},r=1,\ldots ,n_{\text{shuffles}}}{\text{argmin}}\sum_{(i,j)\in M^{\left(r\right)}}{\text{AJSD}}^{\left(r\right)}({\tilde{p}}_{i},{\tilde{q}}_{j}).$$

Random permutations help resolve suboptimal greedy assignments and enforce exclusivity (each source cluster can be matched once per shuffle). This enhances robustness and improves biological plausibility by selecting configurations with clearer inter-cluster separation. We set $n_{\text{trials}}=n_{\text{shuffles}}=25,\tau_{\text{JSD}}=0.45$. The default value of $\tau_{\text{JSD}}$ serves as a practical, empirically robust choice rather than a universal constant. Details (derivations, algorithm, complexity) are in Section B, available as supplementary data at *Bioinformatics* online.

We name our framework *GuidedCoC* to highlight its core mechanism: a *Guided Co-clustering* strategy in which structural information from high-quality unpaired scRNA-seq data informs both the joint co-clustering of cells and features in paired scRNA-seq/scATAC-seq data, and the cross-domain cluster alignment of shared cell populations (see Fig. 1 for an illustrative toy example).

### 2.3 Feature selection and data preprocessing

We begin by selecting highly variable genes (HVGs) from the source scRNA-seq dataset $R^{\left(s\right)}$ using Seurat (Butler *et al.* 2018). To ensure consistency in the feature space, the same set of HVGs is used for the target scRNA-seq dataset $R^{\left(t\right)}$, with homologous genes selected in the case of cross-species integration (homologous genes are mapped using Ensembl BioMart (release 115), retaining only one-to-one orthologs between Mus musculus and Homo sapiens, see details of Example 3 in Section D, available as supplementary data at *Bioinformatics* online). Empirically, a selection of 2500 HVGs is sufficient to capture the key transcriptional variability across cells. Both source and target scRNA-seq datasets are normalized using a ${\;\text{log}\;}_{2}(\cdot +1)$ transformation, applied to UMI counts or TPM values, depending on the data type. For the scATAC-seq modality $A^{\left(t\right)}$, we compute gene activity scores by counting the number of fragments overlapping the gene body and a 2-kb upstream promoter region—following the implementation of the GeneActivity function in the Signac package (Stuart *et al.* 2021). These scores are then log-transformed using ${\;\text{log}\;}_{2}(\cdot +1)$ prior to model input to stabilize variance. Importantly, the conversion of count data into normalized probability distributions [Equation (1)] renders GuidedCoC insensitive to sequencing depth, effectively obviating the need for explicit normalization across modalities.

### 2.4 Evaluation metrics

We assess clustering performance on the target data ($R^{\left(t\right)}$) using two standard metrics: Normalized Mutual Information (NMI) and Adjusted Rand Index (ARI) (Manning *et al.* 2008). Let *Q* be the predicted clustering result and *G* be the ground-truth labels. NMI measures the mutual dependence between *Q* and *G*. Let I(Q;G) be the mutual information, and H(Q) and H(G) be the entropies of *Q* and *G*, respectively. NMI is computed as:


(11) $$\text{NMI}(Q,G)=\frac{I(Q;G)}{\sqrt{H(Q)\cdot H(G)}}.$$

It ranges from 0 (no mutual information) to 1 (perfect correlation). Let $n_{Q,i}$ be the number of samples in the *i*-th cluster of *Q*, $n_{G,j}$ be the number of samples in the *j*-th class of *G*, and $n_{ij}$ be the number of overlapping samples between these two groups. The ARI is computed as:


(12) $$\text{ARI}(Q,G)=\frac{\sum_{ij}\left(\begin{array}{c} n_{ij}\\ 2 \end{array}\right)-\left[\sum_{i}\left(\begin{array}{c} n_{Q,i}\\ 2 \end{array}\right)\sum_{j}\left(\begin{array}{c} n_{G,j}\\ 2 \end{array}\right)\right]/\left(\begin{array}{c} n\\ 2 \end{array}\right)}{\frac{1}{2}\left[\sum_{i}\left(\begin{array}{c} n_{Q,i}\\ 2 \end{array}\right)+\sum_{j}\left(\begin{array}{c} n_{G,j}\\ 2 \end{array}\right)\right]-\left[\sum_{i}\left(\begin{array}{c} n_{Q,i}\\ 2 \end{array}\right)\sum_{j}\left(\begin{array}{c} n_{G,j}\\ 2 \end{array}\right)\right]/\left(\begin{array}{c} n\\ 2 \end{array}\right)}.$$

The ARI adjusts the Rand Index (RI) for chance, with values ranging from -1 (complete mismatch) to 1 (perfect match). Higher values of NMI and ARI indicates better clustering performance.

## 3 Experiments

### 3.1 Datasets

We evaluate on four real-world multiome benchmarks spanning diverse tissues (PBMC, embryonic mouse brain, lymph node/spleen, and pancreatic islets), species (human and mouse), and sequencing platforms, all publicly available with details in Section D, available as supplementary data at *Bioinformatics* online and Table 1. Both source ($R^{\left(s\right)}$) and target ($R^{\left(t\right)}$) datasets provide known cell clusters ($C_{X}$ for $R^{\left(s\right)}$ and ground truth for $R^{\left(t\right)}$ evaluation). For Example 1, we leverage the well-annotated $R^{\left(s\right)}$ to predict $R^{\left(t\right)}$ cell types through cross-domain matching.

**Table 1.** Statistics of unpaired and paired multi-omic single-cell datasets used in our experiments.^a^

Datasets	Unpaired source data ($R^{\left(s\right)}$)	#Cells ($n^{\left(s\right)}$)	#Cell types ($N^{\left(s\right)}$)	Paired target data ($R^{\left(t\right)}/A^{\left(t\right)}$)	#Cells ($n^{\left(t\right)}$)	#Cell types ($N^{\left(t\right)}$)
Example1	scRNA-seq (PBMC, annotated)	2283	7	Multiome (PBMC)	3010	8
Example2	scRNA-seq (E18 MB)	2247	12	Multiome (E18 MB)	3276	9
Example3	scRNA-seq (Mouse LN)	2555	11	Multiome (Human LN)	2426	13
Example4	scRNA-seq (Human PI)	6371	14	Multiome (Human PI)	22 146	8

a PBMC: peripheral blood mononuclear cells; MB: mouse brain; LN: lymph node; PI: pancreatic islet.

### 3.2 Baselines

We evaluate against four state-of-the-art baselines spanning different approaches: MultiVI (Ashuach *et al.* 2023) (deep generative), Cobolt (Gong *et al.* 2021) (generative), scMoMaT (Zhang *et al.* 2023) (matrix factorization), and Seurat v5 (Hao *et al.* 2024) (graph-based). All methods take as input both unpaired and paired datasets (i.e. $R^{\left(s\right)}$, $R^{\left(t\right)}$, and $A^{\left(t\right)}$), employing their standard preprocessing pipelines. The implementation details follow established protocols for joint integration of unpaired and paired single-cell RNA-seq and ATAC-seq data, as described in (Lee *et al.* 2023). We produce these methods’ cell clusters via classical Leiden algorithm (Traag *et al.* 2019) acting on their output embeddings, using ground-truth cluster numbers ($N^{\left(t\right)}$) from the original datasets to ensure fair comparison (consistent with our GuidedCoC implementation). All experiments are conducted on a Linux 64-bit system equipped with an Intel Core i7-14700K (water-cooled), 64 GB DDR5 5600 MHz RAM, an NVIDIA RTX 4090D 24 GB GPU, and a 1250 W power supply.

### 3.3 Experimental results

#### 3.3.1 Performance comparison

**Figure 2.**
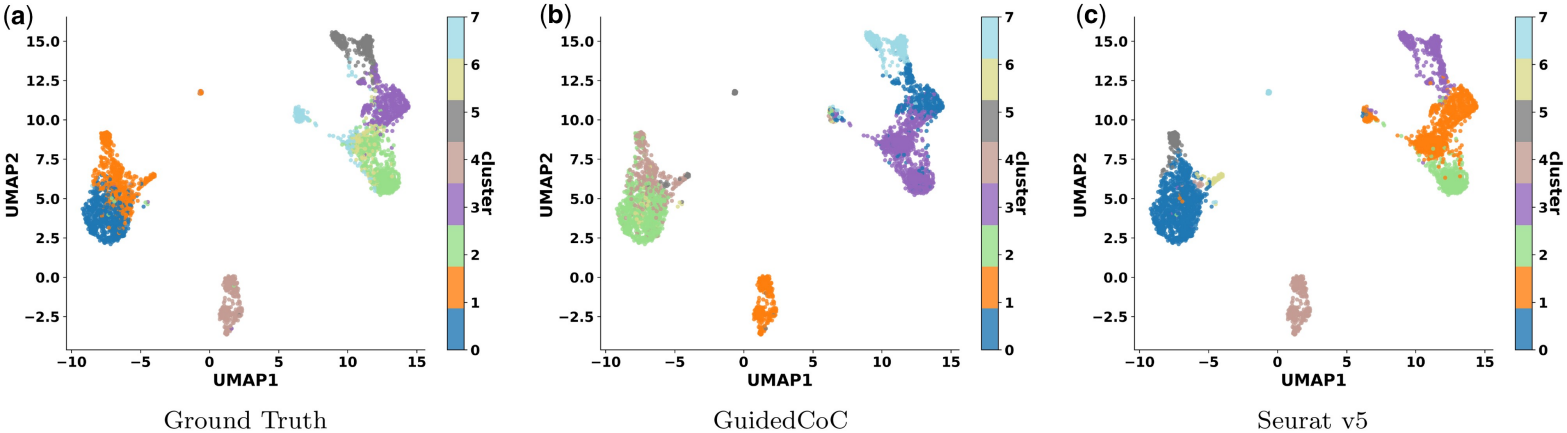
UMAP visualization of cells in the target scRNA-seq dataset $R^{\left(t\right)}$ (Example 1).

To evaluate clustering performance on the target scRNA-seq data $R^{\left(t\right)}$, we conduct extensive experiments comparing GuidedCoC with state-of-the-art baselines (Table 2). Our method demonstrates consistent improvements in NMI and ARI across all benchmarks, particularly in Example 4, attributable to two key advantages: (i) explicit utilization of known cell-population clusters $C_{X}$ from unpaired source data $R^{\left(s\right)}$ (where high-quality cluster structures enable effective knowledge transfer), and (ii) unified feature alignment across $R^{\left(s\right)}$, $R^{\left(t\right)}$, and $A^{\left(t\right)}$ via shared clustering function $C_{Z}$, which jointly addresses cross-domain and cross-modality integration challenges. These synergistic mechanisms collectively explain GuidedCoC’s superior performance in both quantitative metrics and visual assessments (Fig. 2).

**Table 2.** Quantitative comparisons with state-of-the-art approaches across four real-world examples.^a^

Methods	Example 1		Example 2		Example 3		Example 4	
	NMI	ARI	NMI	ARI	NMI	ARI	NMI	ARI
GuidedCoC	**0.6676**	**0.5399**	**0.5640**	**0.4089**	**0.5558**	**0.4539**	**0.7570**	**0.8224**
MultiVI	0.6326	0.5259	0.4928	0.3240	0.4823	0.3542	0.5235	0.3131
Cobolt	0.5420	0.4090	0.4785	0.3392	0.4319	0.2428	0.5847	0.5633
ScMoMaT	0.5259	0.4068	0.4751	0.3269	0.4256	0.2922	0.2572	0.1162
Seurat v5	0.6435	0.4870	0.4928	0.3270	0.5038	0.2624	0.4197	0.1889

a **Bold** indicates best results. Underline indicates second-best results.

#### 3.3.2 UMAP visualization

Figure 2 presents a UMAP visualization of the target scRNA-seq data $R^{\left(t\right)}$ from Example 1, comparing clustering results from GuidedCoC, Seurat v5, and the ground-truth annotations. GuidedCoC successfully captures the major cell populations with improved alignment to the ground truth relative to Seurat v5. Notably, our method preserves the separation between biologically distinct yet spatially adjacent clusters—such as clusters 0 and 1, and clusters 2 and 3 from ground truth annotations—that Seurat v5 tends to partially merge. These results highlight the strength of GuidedCoC in preserving both global separability and local structural consistency during multi-modal integration. More visualization results are available at Section E, available as supplementary data at *Bioinformatics* online.

#### 3.3.3 Ablation study

We conduct a comprehensive ablation study to evaluate the contributions of key components in GuidedCoC (Table 3). Two variants are analyzed: (i) *GuidedCoC w/o* $R^{\left(s\right)}$, which excludes source data by setting β=0 in Equation (7), and (ii) *GuidedCoC w/o* $A^{\left(t\right)}$, which omits auxiliary paired data by setting α=0 in Equation (7). The observed performance degradation in both variants—measured by NMI and ARI—underscores the critical role of external knowledge transfer (via $R^{\left(s\right)}$) and cross-modality integration (via $A^{\left(t\right)}$) for robust multi-omics analysis. This validates our design choices for joint feature and sample alignment.

**Table 3.** Ablation study of GuidedCoC across four examples.^a^

	Example 1		Example 2		Example 3		Example 4	
	NMI	ARI	NMI	ARI	NMI	ARI	NMI	ARI
GuidedCoC	**0.6676**	**0.5399**	**0.5640**	**0.4089**	**0.5558**	**0.4539**	**0.7570**	**0.8224**
GuidedCoC w/o $R^{\left(s\right)}$	0.6478	0.4790	0.5580	0.3948	0.5508	0.4431	0.6827	0.7605
GuidedCoC w/o $A^{\left(t\right)}$	0.6416	0.4679	0.5274	0.3696	0.5382	0.3609	0.7232	0.5375

a **Bold** indicates best results.

#### 3.3.4 Biological interpretation

**Figure 3.**
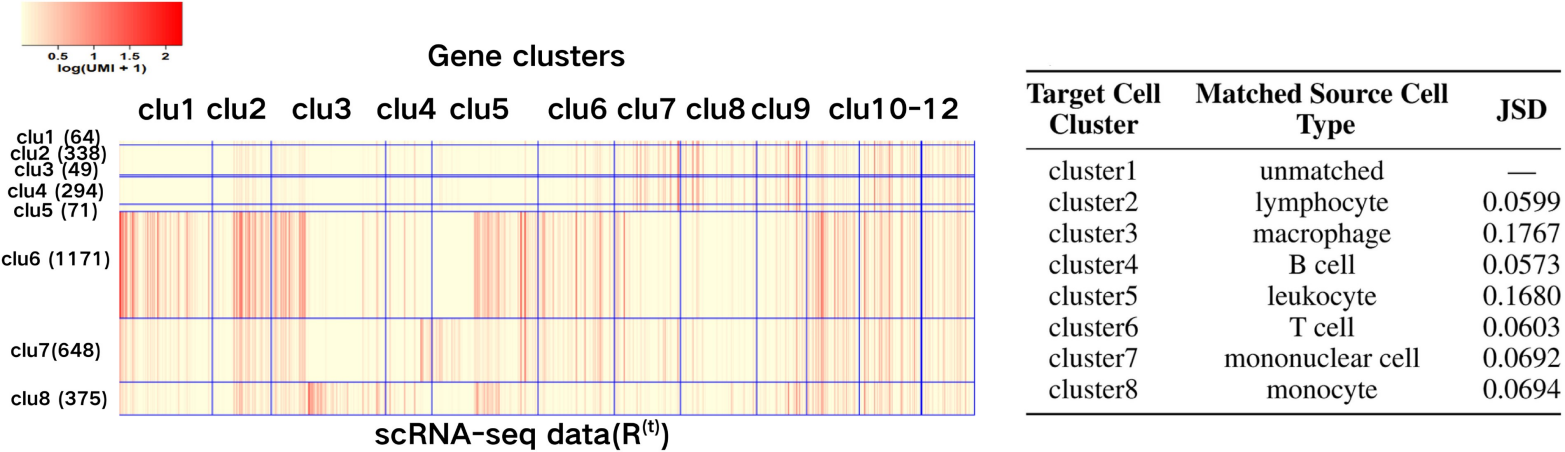
Biological interpretation of GuidedCoC (Example 1). Left: Heatmap of the clustering results for $R^{\left(t\right)}$ (labeled by “clu” for cluster). For clarity, pseudocells are generated by randomly averaging every 20 cells within the same cluster. Right: Cross-domain cell cluster matching results from stage 2 of GuidedCoC. “—” reflects full alignment of all seven source cell types to target clusters, with no residual unmatched populations.

We examine the biological relevance of feature clusters identified by GuidedCoC in Example 1 (Fig. 3), focusing on the three best-matched cell-type pairs based on their top-three Jensen-Shannon divergence (JSD) scores (right panel of Fig. 3). Feature clusters clu1 and clu2 are predominantly associated with cell cluster clu6 in the target scRNA-seq dataset $R^{\left(t\right)}$, which our method matched to T cells in the source dataset $R^{\left(s\right)}$ through cross-domain cell cluster alignment. Additionally, feature clusters clu7–clu9 are enriched in cell clusters clu2 and clu4, corresponding to lymphocytes and B cells in $R^{\left(s\right)}$, respectively. To elucidate the biological functions of these feature clusters, we perform functional enrichment analysis using DAVID (Huang *et al.* 2009a,b). Genes in clu1 show significant enrichment for terms such as *T cell receptor complex* (Bonferroni-corrected *P*-value = $5.68\times 10^{-5}$), reflecting the core structural and signaling components of the TCR. Genes in clu2 are strongly associated with *antigen processing and presentation* and *positive regulation of T cell proliferation* (Bonferroni-corrected *P*-values = $4.51\times 10^{-9}$ and $1.07\times 10^{-6}$, respectively), underscoring their role in T cell activation and immune checkpoint regulation. For full details, see Table A1 in Section F, available as supplementary data at *Bioinformatics* online. Similarly, genes in clu7–clu9 are linked to receptor-mediated activation, cell adhesion, and cytotoxic functions in B cells and lymphocytes (see Table A2 in Section F, available as supplementary data at *Bioinformatics* online).

These results demonstrate that: (i) the integrative co-clustering stage (Stage 1) of our framework effectively groups genes enriched for functional annotation terms that are closely associated with the cell clusters in which they are active; and (ii) the cross-domain clustering alignment stage (Stage 2) accurately matches shared cell types between the source and target datasets. Together, these findings validate the interpretability of our framework and highlight its ability to uncover biologically meaningful relationships between cell types and feature modules.

#### 3.3.5 Sensitivity analysis

**Figure 4.**
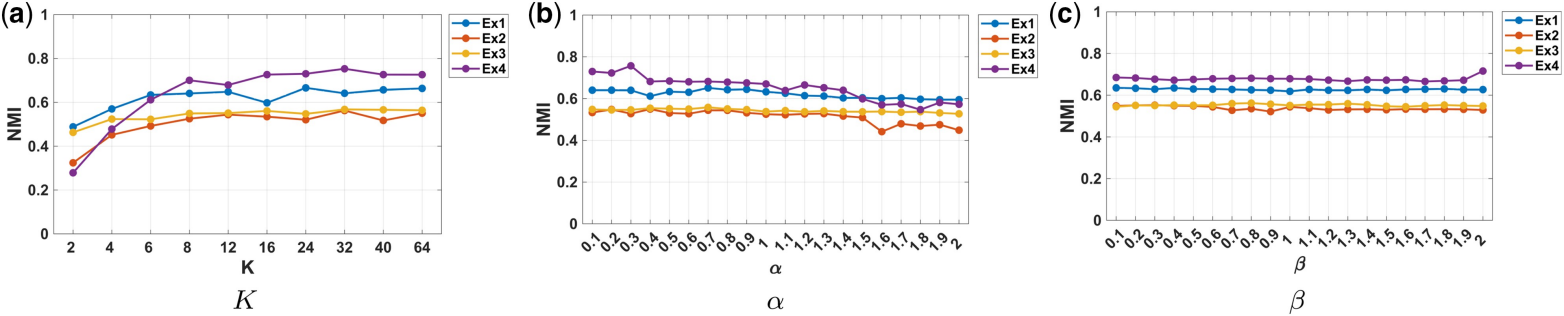
Sensitivity to the number of feature clusters *K* and two weighting factors α and β across four examples.

We evaluate the robustness of GuidedCoC with respect to three key hyperparameters: the number of feature clusters *K*, the modality weighting factor α for the scATAC-seq-derived gene activity/promoter accessibility matrix $A^{\left(t\right)}$, and the domain weighting factor β for the source scRNA-seq data $R^{\left(s\right)}$. Figure 4 reports NMI scores across all four examples under varying hyperparameter values, with others held fixed: (i) Feature cluster count (*K*): Panel (a) shows performance for K∈{2,4,…,64}. NMI improves monotonically until plateauing at K=12, suggesting this value sufficiently captures both modality- and domain-shared structure. We fix K=12 in all experiments. (ii) Modality weight (α): Panel (b) demonstrates stable NMI for α∈(0,1.3], with degradation outside this range. The decline at high α reflects noise in scATAC-seq data; we empirically set α=0.8 to balance information from $A^{\left(t\right)}$. (iii) Domain weight (β): Performance remains robust across β∈(0,2] (Panel (c)), indicating insensitivity to source domain weighting. We use β=1 as default. GuidedCoC exhibits consistent performance across wide hyperparameter ranges, demonstrating its reliability for multi-modal, cross-domain single-cell integration.

#### 3.3.6 Convergence and efficiency

**Figure 5.**
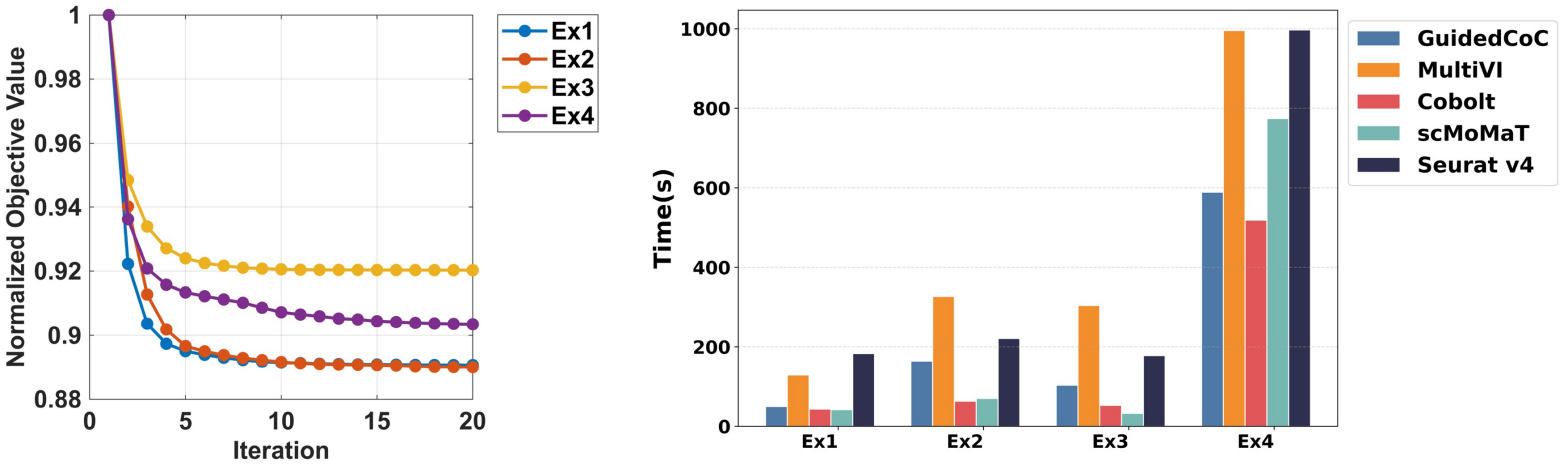
Convergence and computational performance. Left: Loss function convergence curves for GuidedCoC. Right: Running time comparison across datasets.

The left panel of Fig. 5 illustrates the convergence behavior of the normalized objective value over iterations. Across all four examples, the objective decreases rapidly during the initial iterations and stabilizes thereafter, demonstrating both the efficiency and stability of the optimization procedure in GuidedCoC. The right panel of Fig. 5 compares the runtime performance of GuidedCoC with baseline methods. Our method exhibits competitive computational efficiency, particularly when clustering large-scale datasets with approximately 30 000 cells (e.g. Example 4), completing in under 10 minutes and ranking second overall. We attribute this favorable runtime to the use of parallel computing in both stages of our framework (see Section C, available as supplementary data at *Bioinformatics* online, for details on the parallelization strategy).

## 4 Discussion

### 4.1 Choice of source data

The effectiveness of GuidedCoC depends on the selection of a source dataset that is technically robust, biologically relevant, and statistically coherent. Technically, a high-quality source should exhibit strong sequencing metrics, including high UMI counts and a low gene dropout rate. Biologically, it should share major cell populations or conserved functional modules with the target domain, supported by accurate and balanced annotations. Statistically, the source data should possess a clear latent cluster structure, as indicated by high silhouette scores or low entropy in the co-clustering matrix. These properties collectively ensure that the co-clustering objective $L_{R^{\left(s\right)}}(C_{Z}|C_{X})$ transfers biologically meaningful information.

### 4.2 Robustness to feature selection strategy

To ensure our co-clustering framework is not biased by the initial selection of highly variable genes (HVGs) from the source data, we evaluated its performance under multiple feature sets. In addition to our original approach (Case 1: 2500 source-derived HVGs), we constructed three hybrid feature sets by taking the union of HVGs independently selected from both the source and target datasets: Case 2 (5000 HVGs), Case 3 (10 000 HVGs), and Case 4 (15 000 HVGs). Each set comprises an equal number of HVGs from both domains. This design guarantees the inclusion of target-specific genes with high probability. Applied across four benchmark examples, GuidedCoC demonstrates consistent performance under all cases, with marginal differences in normalized mutual information (NMI averages below 2%), see Fig. A4 of Section G, available as supplementary data at *Bioinformatics* online. These results confirm that the method’s co-clustering mechanism, operating through the shared feature clusters $C_{Z}$, robustly identifies target-specific cellular modules even when relying solely on source-derived features.

### 4.3 Cross-domain adaptability

GuidedCoC is explicitly designed to accommodate substantial distribution shifts between source and target domains without assuming strong population overlap. It achieves this through two mechanisms: (i) *Feature-guided structural transfer.* In the Stage-1 co-clustering step [Equation (7)], GuidedCoC transfers knowledge via shared feature clusters ($C_{Z}$) rather than direct cell-level correspondence, capturing conserved transcriptional or regulatory modules (e.g. lineage-specific or pathway-level programs) while allowing target-domain clusters ($C_{Y}$) to adapt independently to condition-specific variation. (ii) *Selective and robust cluster matching.* In Stage-2, cross-domain alignment is based on the Jensen–Shannon divergence (JSD) between cluster-level feature distributions. Only pairs with $\text{JSD}<\tau_{\text{JSD}}=0.45$ are retained as valid matches, while divergent or non-overlapping populations remain unmatched. This selective strategy prevents over-transfer across distinct biological states and has proven effective in partially overlapping scenarios, such as cross-species comparison (Table 1, Example 3).

### 4.4 Assumption robustness

GuidedCoC relies on a mild and biologically plausible assumption that a subset of target features (e.g. gene activity or promoter accessibility) is not only linked to $R^{\left(t\right)}$ but also exhibits partially shared clustering structures with the source scRNA-seq data $R^{\left(s\right)}$. This does not require one-to-one or fully conserved structures across domains—it only assumes that certain transcriptional programs or regulatory modules remain consistent. When such structural similarity weakens or does not hold (e.g. in cross-species or disease-perturbed settings), GuidedCoC degrades gracefully.

To empirically assess the assumption’s impact, we performed two additional experiments. (i) Fixing $R^{\left(t\right)}$ while randomly reordering the features of $A^{\left(t\right)}$ to disrupt its relationship with $R^{\left(t\right)}$ resulted in only mild performance drops, moderated by the modality-weighting factor α (Table A3, available as supplementary data at *Bioinformatics* online). (ii) Fixing the target data in Example 1 but replacing the source data with those from Examples 2–4 to remove shared clustering structures yielded NMI values of 0.5846, 0.6233, and 0.5806, moderately lower than the original 0.6676. Empirically, the influence of the source data is modulated by the domain-weighting factor β [Equation (7)]. When source-target correspondence is low, the model degrades gracefully, converging toward the target-only baseline (see Table 3). These results confirm that GuidedCoC maintains stable performance even when the shared structure is weakened, consistent with its design for robust, selective cross-domain transfer.

### 4.5 Evaluation of unsupervised label transfer performance

Stage 2 of GuidedCoC can be interpreted as performing unsupervised label transfer through cross-domain cluster matching. To quantitatively evaluate this capability, we conducted additional experiments on Example 1, where ground truth labels are available for the source domain but not for the target. We derived reference annotations for target cells using two independent methods: Seurat (Setting 1) and SingleR (Aran *et al.* 2019) (Setting 2). These annotated datasets served as proxy ground truth for assessing label-transfer accuracy. Our method was compared against two established integration approaches, scNCL (Yan *et al.* 2023) and scJoint (Lin *et al.* 2022), using both Weighted F1 and Macro F1 scores. As shown in Table A4, available as supplementary data at *Bioinformatics* online, our method achieves competitive, and in Setting 1 superior label-transfer accuracy compared to the benchmark methods, validating its effectiveness in cross-domain cell-type annotation.

## 5 Conclusion

We have presented *GuidedCoC*, a novel framework for integrative analysis of paired single-cell RNA-seq (scRNA-seq) and ATAC-seq (scATAC-seq) data guided by unpaired reference scRNA-seq datasets, addressing the critical challenge of cross-modal knowledge transfer in single-cell multi-omics analysis. By jointly co-clustering cells and features across modalities and domains through an information-theoretic objective, GuidedCoC simultaneously (i) aligns gene expression and chromatin accessibility patterns, (ii) enhances cell-type resolution via cross-modal regularization, and (iii) automatically identifies shared cell populations between source and target datasets. Empirical results demonstrate that GuidedCoC consistently outperforms state-of-the-art methods in clustering accuracy while achieving superior biological interpretability and computational scalability, establishing structural transfer as a powerful approach for robust single-cell data integration.

Our framework offers a scalable and annotation-free approach to multi-modal data integration, particularly valuable when high-quality paired measurements are limited. GuidedCoC can empower downstream biological discovery by improving cell-type resolution and uncovering interpretable feature modules linked across omics layers. More broadly, the idea of leveraging structural priors from large, unpaired datasets opens new opportunities for integrating diverse data modalities in other domains such as spatial transcriptomics or single-cell proteogenomics. As single-cell atlases continue to grow, methods like GuidedCoC that leverage their structure without requiring explicit labels are increasingly impactful.

Despite its promising performance, GuidedCoC assumes the availability of a high-quality source dataset and partial overlap between cell populations in source and target datasets. Moreover, the current framework focuses on gene activity scores/promoter accessibility as the links between modalities; extending it to accommodate more complex regulatory relationships (e.g. enhancer-promoter interactions or TF binding motifs) is an important direction for future research. Finally, formalizing theoretical guarantees for transferability and extending the approach to support more than two omics layers would further enhance its utility in comprehensive single-cell multi-omics integration.

## Supplementary Material

btaf639_Supplementary_Data

## Data Availability

All data generated or analysed during this study are included in this published article and its supplementary materials.
