# Supplementary material for: Guided co-clustering transfer across unpaired and paired single-cell multi-omics data
Source: Bioinformatics. 2025 Dec 1;41(12):btaf639. doi: 10.1093/bioinformatics/btaf639 (PMC12696646; doi:10.1093/bioinformatics/btaf639)
Supplement: btaf639_Supplementary_Data [file btaf639_supplementary_data.zip › GuidedCoC_Supplementary_Materials.pdf]

Supplementary Materials for "Guided Co-clustering Transfer Across Unpaired and Paired Single-cell Multi-omics Data"

Hongyao Li[1,†], Yunrui Liu[1,†], and Pengcheng Zeng[1,*]

[1]Institute of Mathematical Sciences, ShanghaiTech University, Shanghai, China

November 21, 2025

A Optimization for Integrative Co-clustering

A.1 Mathematical Derivation

The goal is to jointly infer the cluster assignments C_Y (target domain cell clusters) and C_Z (feature clusters) by minimizing the divergence between the observed joint distributions and their cluster-induced approximations. Specifically, we solve the following optimization problem:

$$\begin{aligned}\min_{C_Y, C_Z} \quad & D_{\mathrm{KL}}\left(\mathbb{P}_{\mathcal{R}^{(t)}}(Y,Z) \,\|\, \mathbb{P}^*_{\mathcal{R}^{(t)}}(Y,Z)\right) + \alpha \cdot D_{\mathrm{KL}}\left(\mathbb{P}_{\mathcal{A}^{(t)}}(Y,Z) \,\|\, \mathbb{P}^*_{\mathcal{A}^{(t)}}(Y,Z)\right) \\ & + \beta \cdot D_{\mathrm{KL}}\left(\mathbb{P}_{\mathcal{R}^{(s)}}(X,Z) \,\|\, \mathbb{P}^*_{\mathcal{R}^{(s)}}(X,Z)\right), \end{aligned} \tag{A1}$$

where α and β are tunable trade-off parameters. Each KL divergence term quantifies how well the learned clustering structure captures the joint dependency of variables in different data modalities or domains.

To minimize this objective, we adopt an alternating optimization strategy by iteratively updating C_Y and C_Z, while keeping the other fixed.

1. **Fix C_X:** The cell type labels in the source domain $\mathcal{R}^{(s)}$ are assumed known and fixed, so C_X is not updated during optimization.

2. **Update C_Y given C_Z:** With feature clusters C_Z fixed, we update the cell clustering in the target domain by minimizing the following objective:

$$\sum_{i=1}^{N^{(t)}} \sum_{y \in \{y: C_Y(y)=i\}} \mathbb{P}_{\mathcal{R}^{(t)}}(Y=y) \cdot Q(\tilde{Y}=i, Y=y \mid C_Z),$$

† These authors contributed equally.

∗ Corresponding author: zengpch@shanghaitech.edu.cn

where the per-sample cost function $Q(\tilde{Y}, Y \mid C_Z)$ is defined as:

$$
\begin{aligned}
Q(\tilde{Y} = i, Y = y \mid C_Z) \triangleq\, & D_{\mathrm{KL}}(\mathbb{P}_{\mathcal{R}^{(t)}}(Z \mid Y = y) \,\|\, \mathbb{P}^*_{\mathcal{R}^{(t)}}(Z \mid \tilde{Y} = i, Y = y)) \\
& + \alpha \cdot \frac{\mathbb{P}_{\mathcal{A}^{(t)}}(Y = y)}{\mathbb{P}_{\mathcal{R}^{(t)}}(Y = y)} \cdot D_{\mathrm{KL}}(\mathbb{P}_{\mathcal{A}^{(t)}}(Z \mid Y = y) \,\|\, \mathbb{P}^*_{\mathcal{A}^{(t)}}(Z \mid \tilde{Y} = i, Y = y)).
\end{aligned}
$$

Each cell $y \in \mathcal{R}^{(t)}$ is assigned to the cluster i that minimizes the local cost:

$$
C_Y(y) = \arg \min_{i \in \{1,\ldots,N^{(t)}\}} Q(\tilde{Y} = i, Y = y \mid C_Z). \tag{A2}
$$

3. **Update C_Z given C_Y:** With the target cell clusters fixed, we next update the feature clusters by minimizing:

$$
\sum_{j=1}^{K} \sum_{z \in \{z : C_Z(z) = j\}} R(\tilde{Z} = j, Z = z \mid C_Y),
$$

where the function $R(\tilde{Z}, Z \mid C_Y)$ aggregates divergence costs across domains:

$$
\begin{aligned}
R(\tilde{Z} = j, Z = z \mid C_Y) \triangleq\, & \mathbb{P}_{\mathcal{R}^{(t)}}(Z = z) \cdot D_{\mathrm{KL}}(\mathbb{P}_{\mathcal{R}^{(t)}}(Y \mid Z = z) \,\|\, \mathbb{P}^*_{\mathcal{R}^{(t)}}(Y \mid \tilde{Z} = j, Z = z)) \\
& + \alpha \cdot \mathbb{P}_{\mathcal{A}^{(t)}}(Z = z) \cdot D_{\mathrm{KL}}(\mathbb{P}_{\mathcal{A}^{(t)}}(X \mid Z = z) \,\|\, \mathbb{P}^*_{\mathcal{A}^{(t)}}(Y \mid \tilde{Z} = j, Z = z)) \\
& + \beta \cdot \mathbb{P}_{\mathcal{R}^{(s)}}(Z = z) \cdot D_{\mathrm{KL}}(\mathbb{P}_{\mathcal{R}^{(s)}}(X \mid Z = z) \,\|\, \mathbb{P}^*_{\mathcal{R}^{(s)}}(X \mid \tilde{Z} = j, Z = z)).
\end{aligned}
$$

Each feature $z \in \{1, \ldots, q\}$ is assigned to the cluster s that minimizes the above expression:

$$
C_Z(z) = \arg \min_{j \in \{1,\ldots,K\}} R(\tilde{Z} = j, Z = z \mid C_Y). \tag{A3}
$$

A.2 Integrative Co-Clustering Algorithm

We provide the detailed algorithm of the optimization for integrative co-clustering in Algorithm 1.

Algorithm 1 Integrative Co-Clustering

Require: Joint distributions: $\mathbb{P}_{\mathcal{R}^{(t)}}, \mathbb{P}_{\mathcal{A}^{(t)}}, \mathbb{P}_{\mathcal{R}^{(s)}}$; trade-off parameters α, β; number of clusters $N^{(t)}$, K.

Ensure: Cluster assignments: C_Y (target cells), C_Z (features)

Initialize C_Y and C_Z randomly
repeat
  Update C_Y given C_Z:
  for each cell $y \in \{1, \ldots, n^{(t)}\}$ **do**
    for each cluster $i \in \{1, \ldots, N^{(t)}\}$ **do**
      Compute $Q(\tilde{Y} = i, Y = y \mid C_Z)$ using Eq. (A2)
    end for
    Assign $C_Y(y) \leftarrow \arg\min_i Q(\tilde{Y} = i, Y = y \mid C_Z)$
  end for
  Update C_Z given C_Y:
  for each feature $z \in \{1, \ldots, q\}$ **do**
    for each cluster $j \in \{1, \ldots, K\}$ **do**
      Compute $R(\tilde{Z} = j, Z = z \mid C_Y)$ using Eq. (A3)
    end for
    Assign $C_Z(z) \leftarrow \arg\min_j R(\tilde{Z} = j, Z = z \mid C_Y)$
  end for
until convergence
return C_Y, C_Z

Complexity of Algorithm 1. Here we consider per-iteration cost. Let $n^{(t)} = \#row\ of\ \mathcal{R}^{(t)}$, $n^{(s)} = \#row\ of\ \mathcal{R}^{(s)}$, $q = \#features$, $N^{(t)} = \#cell - clusters$, and $K = \#feature - clusters$.

$$\begin{aligned} &\text{Update } C_Y: \quad \mathcal{O}\Big(n^{(t)} \cdot N^{(t)} \cdot q\Big), \\ &\text{Update } C_Z: \quad \mathcal{O}\Big(q \cdot K \cdot (n^{(s)} + n^{(t)})\Big), \end{aligned} \quad \Longrightarrow \quad \mathcal{O}\Big(q\,(N^{(t)}n^{(t)} + Kn^{(s)} + Kn^{(t)})\Big).$$

The calculation of complexity has ignored the multiples of constants, and the complexity of calculating $\mathbb{P}^*_{\mathcal{R}^{(t)}}$, $\mathbb{P}^*_{\mathcal{R}^{(s)}}$ and $\mathbb{P}^*_{\mathcal{A}^{(t)}}$ has been omitted (in the process of calculating Q, and the complexity is $\mathcal{O}((n^{(s)} + n^{(t)})q)$, so it is not included in the total complexity).

A.3 Convergence

The following theorem shows that the Algorithm 1 converges to a coordinate-wise stationary point. It indicates the algorithm will converge to a local minimum. Finding the global optimal solution is NP-hard.

Theorem A.1. *Let $F(C_Y, C_Z) = \mathcal{L}_{\mathcal{R}^{(t)}}(C_Y, C_Z) + \alpha\,\mathcal{L}_{\mathcal{A}^{(t)}}(C_Y, C_Z) + \beta\,\mathcal{L}_{\mathcal{R}^{(s)}}(C_Z \mid C_X)$ and assume each divergence is finite. Then the alternating updates*

$$C_Y^{(t+1)} \;=\; \arg\min_{C_Y} F(C_Y,\, C_Z^{(t)}), \qquad C_Z^{(t+1)} \;=\; \arg\min_{C_Z} F(C_Y^{(t+1)},\, C_Z)$$

generate a sequence $\{F(C_Y^{(t)}, C_Z^{(t)})\}_{t\geq 0}$ *that is* monotone nonincreasing, lower-bounded *by zero, and converges to a limit value* F^*. *Moreover, any limit point* (C_Y^*, C_Z^*) *of the sequence of assignments satisfies the coordinate-wise optimality conditions*

$$C_Y^* \;=\; \arg\min_{C_Y} F(C_Y,\, C_Z^*), \quad C_Z^* \;=\; \arg\min_{C_Z} F(C_Y^*,\, C_Z),$$

i.e. is a coordinate-wise stationary point *of* F.

Proof. We break the proof into three parts.

(a) Monotonicity. At iteration t, first update

$$C_Y^{(t+1)} = \arg\min_{C_Y} F\Big(C_Y,\, C_Z^{(t)}\Big).$$

By choosing the global minimizer in C_Y (over a finite discrete set) we have

$$F\Big(C_Y^{(t+1)},\, C_Z^{(t)}\Big) \;\leq\; F\Big(C_Y^{(t)},\, C_Z^{(t)}\Big).$$

Next, with C_Y fixed, update

$$C_Z^{(t+1)} = \arg\min_{C_Z} F\Big(C_Y^{(t+1)},\, C_Z\Big),$$

which similarly yields

$$F\Big(C_Y^{(t+1)},\, C_Z^{(t+1)}\Big) \;\leq\; F\Big(C_Y^{(t+1)},\, C_Z^{(t)}\Big).$$

Combining the two inequalities establishes

$$F\Big(C_Y^{(t+1)},\, C_Z^{(t+1)}\Big) \;\leq\; F\Big(C_Y^{(t)},\, C_Z^{(t)}\Big),$$

so the sequence $\{F(C_Y^{(t)}, C_Z^{(t)})\}$ is monotone nonincreasing.

(b) Lower-Boundedness. Each KL divergence $D_{\mathrm{KL}}(\cdot\|\cdot)$ is nonnegative and finite by assumption, hence $F(C_Y, C_Z) \geq 0$ for all assignments. Thus the monotone nonincreasing sequence is bounded below by zero.

(c) Convergence and Stationarity. A real sequence that is monotone nonincreasing and bounded below converges to a finite limit F^*. Denote by $\{(C_Y^{(t_k)}, C_Z^{(t_k)})\}_{k\geq 1}$ a subsequence converging to some limit point (C_Y^*, C_Z^*) (which exists because the assignment sets are finite).

We now show that (C_Y^*, C_Z^*) satisfies the coordinate-wise optimality conditions. By continuity of F in its discrete arguments (i.e. it takes only finitely many values), the limiting arguments satisfy

$$F\Big(C_Y^*,\, C_Z^*\Big) = \lim_{k\to\infty} F\Big(C_Y^{(t_k)},\, C_Z^{(t_k)}\Big)$$

and, since each $C_Y^{(t_k+1)}$ minimized $F(\,\cdot\,, C_Z^{(t_k)})$,

$$F\Big(C_Y^*,\, C_Z^*\Big) \;\leq\; F\Big(C_Y,\, C_Z^*\Big) \quad \text{for all } C_Y.$$

Similarly, from the minimization in the C_Z-update,

$$F\Big(C_Y^*,\, C_Z^*\Big) \;\le\; F\Big(C_Y^*,\, C_Z\Big) \quad \text{for all } C_Z.$$

These two inequalities jointly imply that (C_Y^*, C_Z^*) is a coordinate-wise stationary point:

$$C_Y^* = \arg\min_{C_Y} F(C_Y,\, C_Z^*), \quad C_Z^* = \arg\min_{C_Z} F(C_Y^*,\, C_Z).$$

This concludes the proof. □

B Details for Cross-Domain Cluster Matching

B.1 Derivation

Let C_X denote the known cell type labels in the source domain $\mathcal{R}^{(s)}$, and let C_Y represent the clustering results inferred in the target domain $\mathcal{R}^{(t)}$. We assume that a subset of clusters in C_X and C_Y correspond to shared biological cell types across domains. Our goal is to establish one-to-one correspondences between such clusters, enabling accurate label transfer from $\mathcal{R}^{(s)}$ to $\mathcal{R}^{(t)}$.

Let $\tilde{p}_i$ and $\tilde{q}_j$ denote the cluster-level feature profiles derived from the i-th row of $\widetilde{\mathbb{P}}_{\mathcal{R}^{(s)}}(\tilde{Y}, \tilde{Z})$ and the j-th row of $\widetilde{\mathbb{P}}_{\mathcal{R}^{(t)}}(\tilde{X}, \tilde{Z})$, respectively. Specifically, for each cluster $i \in C_X$, we extract the corresponding rows of $\widetilde{\mathbb{P}}_{\mathcal{R}^{(s)}}$ to obtain $\tilde{p}_i$; analogously, $\tilde{q}_j$ is constructed from rows in $\widetilde{\mathbb{P}}_{\mathcal{R}^{(t)}}$ associated with cluster $j \in C_Y$.

To identify matched clusters, we consider n_{shuffles} random permutations of the target cluster labels in C_Y, indexed by $r = 1, \ldots, n_{\text{shuffles}}$. For each permutation r, clusters are matched in sequence using a greedy strategy that minimizes the *average Jensen-Shannon divergence* (AJSD):

$$\text{AJSD}^{(r)}(\tilde{p}_i, \tilde{q}_j) = \frac{1}{n_{\text{trials}}} \sum_{l=1}^{n_{\text{trials}}} \text{JSD}^{(l)}(\tilde{p}_i, \tilde{q}_j),$$

where in each trial l, equal-sized subsets are sampled from $\tilde{p}_i$ and $\tilde{q}_j$ to mitigate potential imbalance between source and target clusters.

The **Jensen-Shannon divergence** between two discrete distributions P and Q is defined as:

$$\text{JSD}(P \parallel Q) = \frac{1}{2} D_{\text{KL}}(P \parallel M) + \frac{1}{2} D_{\text{KL}}(Q \parallel M), \quad \text{where } M = \frac{1}{2}(P + Q),$$

and $D_{\text{KL}}(\cdot \parallel \cdot)$ denotes the Kullback-Leibler divergence.

For a fixed target cluster j, we select the best matching source cluster k by minimizing the AJSD:

$$k = \underset{i=1,\ldots,N^{(s)}}{\text{argmin}} \; \text{AJSD}^{(r)}(\tilde{p}_i, \tilde{q}_j).$$

The match (k, j) is accepted if $\text{AJSD}^{(r)}(\tilde{p}_k, \tilde{q}_j) < \tau_{\text{JSD}}$, where τ_{JSD} is a predefined threshold (default: 0.45). If this condition is not satisfied, $\tilde{q}_j$ is considered unmatched—potentially corresponding to a novel cell type not observed in the source domain.

Within each permutation r, once a source cluster has been matched, it is excluded from further assignments in that permutation, enforcing an exclusive one-to-one matching constraint.

This matching process is repeated across n_{shuffles} permutations of C_Y, producing a set of candidate matchings $\{\mathcal{M}^{(r)}\}_{r=1}^{n_{\text{shuffles}}}$. To determine the optimal matching configuration, we select the permutation r^* that minimizes the total divergence among all valid matches:

$$\mathcal{M}^* = \underset{\mathcal{M}^{(r)}, r=1,\ldots,n_{\text{shuffles}}}{\text{argmin}} \sum_{(k,j)\in\mathcal{M}^{(r)}} \text{AJSD}^{(\text{r})}(\tilde{p}_k, \tilde{q}_j),$$

where the summation is restricted to matched pairs satisfying the AJSD threshold.

Rationale for Permutation-Based Matching. The permutation index r denotes an ordering of target clusters in C_Y, e.g., $[1,2,3,4,5]$ vs. $[2,3,4,5,1]$. Multiple permutations are evaluated for two key reasons: (i) *Exclusive Matching Enforcement* — once a source cluster is assigned, it cannot be reused in the same permutation, avoiding duplicate matches; (ii) *Optimization of Match Quality* — a fixed order may yield suboptimal results due to greedy assignments. For example, reordering clusters may better align with biological structure and minimize divergence. Therefore, we select the configuration that maximizes aggregate JSD, reflecting more separable and interpretable cluster correspondences.

In summary, the selected matching configuration $\mathcal{M}^*$ optimizes the divergence between matched clusters under exclusivity constraints, yielding high-confidence mappings that facilitate accurate and biologically meaningful label transfer.

B.2 Algorithm

We provide the detailed algorithm of permutation-based matching across source and target clusters in Algorithm 2.

Complexity of Algorithm 2 (Permutation-Based Matching). Let $N^{(t)}, N^{(s)}$ be #target and #source clusters, $T = n_{\text{trials}}$, and $R = n_{\text{shuffles}}$, with profile dimension m.

$$\text{Cost} \;=\; \mathcal{O}\Big(R \cdot N^{(t)} \cdot N^{(s)} \cdot T \cdot m \cdot c\Big)\,.$$

The parameter c represents the average number of rows utilized for Jensen-Shannon divergence (JSD) computations in each matching trial. This value is data-dependent, defined as $c = mean \sum_{i=1}^{N^{(t)}} \sum_{j=1}^{N^{(s)}} c_{ij}$, where $c_{ij} = min(nr_i, nr_j)$, where nr_i and nr_j denote the row counts of i-th cluster of target data and j-th cluster of source data, respectively. The computational complexity for JSD calculation in a single trial is $O(mn_{trials}c)$. In worst-case scenarios, where all rows of either the source or target data might be processed, the theoretical time complexity of the matching algorithm scales as:

$$\text{Cost} \;= \mathcal{O}\Big(R \cdot T \cdot m \cdot min(n^{(s)}N^{(t)}, n^{(t)}N^{(s)})\Big)\,.$$

However, the practical complexity is reduced because previously matched clusters are excluded in subsequent iterations.

Notably, the matching algorithm's time complexity is comparable to that of the CoCluster algorithm. Consequently, optimizing runtime efficiency necessitates balanced parameter configuration for both algorithms.

Algorithm 2 Permutation-Based Matching

Require: Cluster-level profiles $\{\tilde{p}_i\}_{i=1}^{N^{(s)}}, \{\tilde{q}_j\}_{j=1}^{N^{(t)}}$; number of permutations n_{shuffles}; number of trials n_{trials}; threshold τ_{JSD}

Ensure: Optimal matching configuration $\mathcal{M}^*$

for $r = 1$ to n_{shuffles} **do**
  Randomly permute order of target clusters C_Y
  Initialize $\mathcal{M}^{(r)} \leftarrow \emptyset$, used source clusters $\leftarrow \emptyset$
  for each target cluster j in permuted C_Y **do**
    for each source cluster $i \notin$ used source clusters **do**
      Estimate $\text{AJSD}^{(r)}(\tilde{p}_i, \tilde{q}_j)$ via n_{trials} bootstrap samples
    end for
    Let $k \leftarrow \arg\min_i \text{AJSD}^{(r)}(\tilde{p}_i, \tilde{q}_j)$
    if $\text{AJSD}^{(r)}(\tilde{p}_k, \tilde{q}_j) < \tau_{\text{JSD}}$ **then**
      Add (k, j) to $\mathcal{M}^{(r)}$; add k to used source clusters
    end if
  end for
end for
Select $r^* = \arg\min_r \sum_{(i,j)\in\mathcal{M}^{(r)}} \text{AJSD}^{(r)}(\tilde{p}_i, \tilde{q}_j)$
return $\mathcal{M}^* = \mathcal{M}^{(r^*)}$

C Parallel Computing Analysis for Algorithms 1 & 2

The co-clustering algorithm exhibits inherent parallelization potential during computation of the objective function Q, which proceeds in two distinct stages. First, the adjusted distribution $\mathbb{P}^*$ derived from clustering results C_Y and C_Z can be computed in parallel across cell (Y) or feature (Z) dimensions since $\mathbb{P}^*$ is uniquely determined by these indices without data competition. Second, the Q function update using fixed $\mathbb{P}$ and $\mathbb{P}^*$ allows parallel execution across either Y/Z dimensions (depending on which is being updated) or cluster labels i, as each computation depends only on the current cell/feature and cluster assignment. These two layers of parallelism could theoretically achieve 70-80% parallel operations (yielding 2.3-2.9× speedup on 4-core systems in our experiments).

Similarly, the matching function permits parallelization as both permutation operations (mutually independent) and Jensen-Shannon Divergence (JSD) calculations (stateless and non-iterative) can be distributed across threads. However, nested implementation introduces substantial scheduling overhead and faces limitations in environments like MATLAB lacking hierarchical parallelization support. Consequently, GUIDEDCOC implements single-level parallelism restricted to JSD computation.

D Dataset Details

We provide detailed descriptions of the four benchmark datasets used in our experiments:

- **Example 1 – PBMC Human Data:** The unpaired scRNA-seq data (3,709 cells) was collected

from a healthy male donor (age 18–35) using the Chromium GEM-X Single Cell 3' v4 platform (*10X Genomics*). This dataset is available at `https://www.10xgenomics.com/datasets/5k_Human_Donor1_PBMC_3p_gem-x`. The paired scRNA-seq + scATAC-seq multiome data (3,012 cells) was obtained from a healthy female donor using the Chromium Single Cell Multiome ATAC + Gene Expression platform. The datasets are available at `https://www.10xgenomics.com/datasets/pbmc-from-a-healthy-donor-no-cell-sorting-3-k-1-standard-1-0-0`.

- **Example 2 – E18 Mouse Brain:** The unpaired scRNA-seq data (11,843 cells) was derived from the cortex, hippocampus, and subventricular zone of an embryonic day 18 (E18) mouse brain using Chromium v3 chemistry. This dataset is publicly available at `https://www.10xgenomics.com/datasets/10-k-brain-cells-from-an-e-18-mouse-v-3-chemistry-3-standard-3-0-0`.The paired multiome data (4,481 cells) was generated from fresh E18 brain tissue using the ATAC + RNA multiome protocol. The datasets are available at `https://www.10xgenomics.com/datasets/fresh-embryonic-e-18-mouse-brain-5-k-1-standard-1-0-0`.

- **Example 3 – Mouse and Human Lymph Node:** The unpaired scRNA-seq data was collected from spleen and lymph nodes of an 8-month-old C57BL/6J mouse using the TotalSeq-C Mouse Universal Cocktail. We used the "Lymph node rep 2" sample. The dataset is publicly available at `https://www.10xgenomics.com/datasets/Mixture-of-cells-from-mouse-lymph-nodes-and-spleen-stained-with-totalseqc-mouse-universal-cocktail`. The paired dataset consists of human lymph node nuclei (14,645 high-quality nuclei) from a patient with diffuse small B-cell lymphoma, profiled using the Multiome ATAC + RNA platform. The datasets are available at `https://www.10xgenomics.com/datasets/fresh-frozen-lymph-node-with-b-cell-lymphoma-14-k-sorted-nuclei-1-standard-1-0-0`. We obtained high-confidence human-mouse orthologous gene pairs using the BioMart tool (release 115, `https://asia.ensembl.org`) in the Ensembl database (GRCh38.p13). Human gene identifiers and their murine orthologs were retrieved by selecting one-to-one orthology relationships from the "Homologues" section. Orthology predictions in Ensembl are based on gene tree analysis of protein sequences across species, with confidence scores assigned to ensure reliability; only high-confidence pairs were retained for downstream analysis.

- **Example 4 – Pancreatic Islet:** The unpaired scRNA-seq data comes from human pancreatic tissues and is part of the GSE84133 dataset, which includes multiple donors and tissue types. The dataset is available at `https://www.ncbi.nlm.nih.gov/geo/query/acc.cgi?acc=GSE84133`. The paired multiome data was collected from healthy donors in the GSE200044 project, which profiled over 85,000 human islet cells from donors with various diabetic statuses. Only healthy donor cells were used in our analysis [Chiou et al., 2023]. The dataset can be accessed at `https://www.ncbi.nlm.nih.gov/geo/query/acc.cgi?acc=GSE200044`.

E More UMAP Visualizations

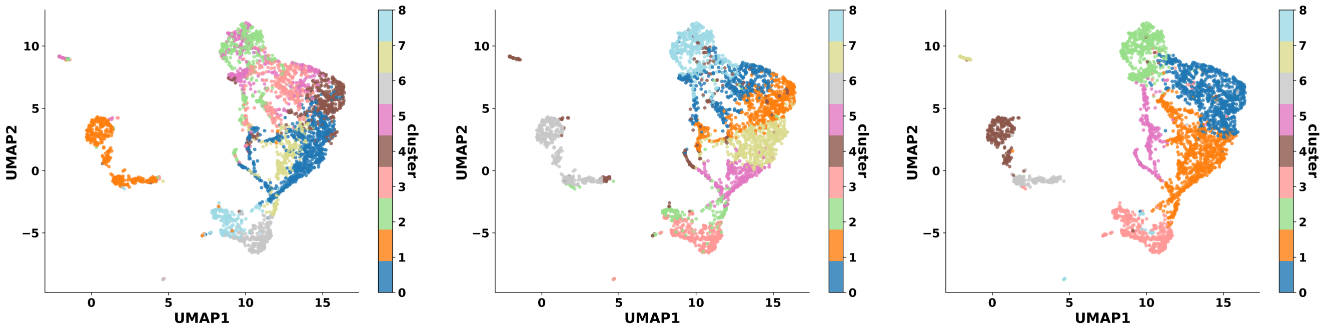

(a) Ground Truth (b) GuidedCoC (c) Seurat v5

Figure A1: UMAP visualization of cells in the target scRNA-seq dataset $\mathcal{R}^{(t)}$ (Example 2).

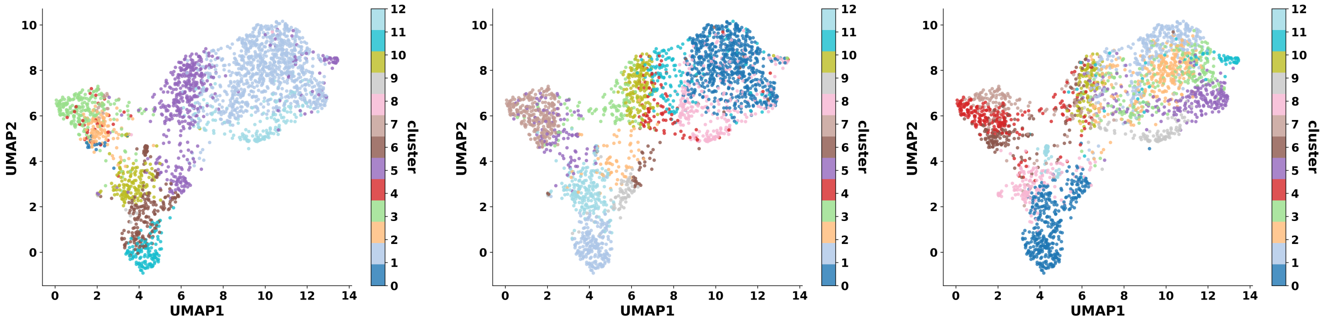

(a) Ground Truth (b) GuidedCoC (c) Seurat v5

Figure A2: UMAP visualization of cells in the target scRNA-seq dataset $\mathcal{R}^{(t)}$ (Example 3).

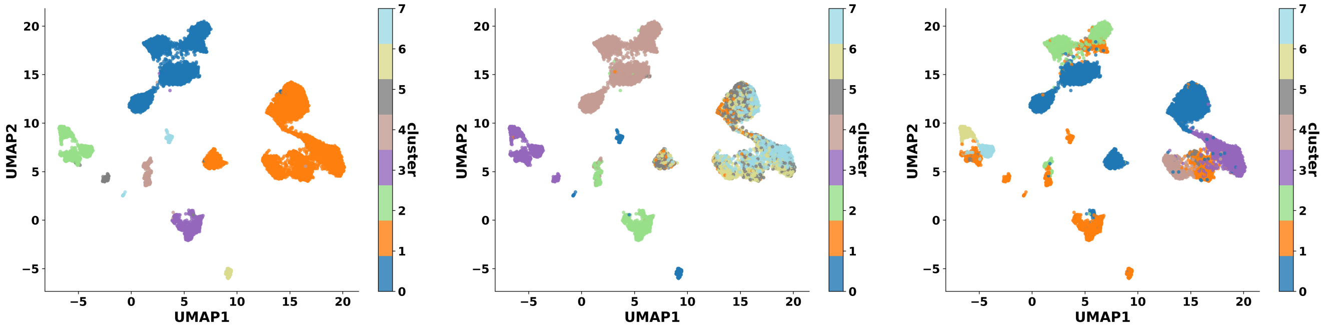

(a) Ground Truth (b) GuidedCoC (c) Seurat v5

Figure A3: UMAP visualization of cells in the target scRNA-seq dataset $\mathcal{R}^{(t)}$ (Example 4).

F Details of Enrichment Analysis

Table A1: Selected enriched annotation terms for the gene lists in **Feature Clusters 1 and 2** of Example 1. Cluster 1 includes canonical T cell receptor genes such as *TRBV18*, *TRBC2*, *CD8A*, *CD8B*, and *CD247*; Cluster 2 contains regulatory genes including *CD70*, *IL7*, *HLA-DPB1*, and *PDCD1LG2*.

**Category**	**Term**	**Count**	**%**	**Bonferroni P-value**
GOTERM_BP_DIRECT	Antigen processing and presentation	11	5.64	4.51×10^{-9}
UP_KW_CELLULAR_COMPONENT	T cell receptor	14	3.44	2.76×10^{-5}
GOTERM_BP_DIRECT	Positive regulation of T cell activation	9	4.62	1.07×10^{-6}
GOTERM_CC_DIRECT	T cell receptor complex	15	3.69	5.68×10^{-5}
GOTERM_BP_DIRECT	Adaptive immune response	23	5.65	4.87×10^{-2}

Table A2: Selected enriched annotation terms for Feature Clusters 7–9. The gene lists include representative genes associated with immune functions such as *FCGR2B*, *DLG2*, *FCGR3A*, and the top pathways are shown.

**Category**	**Term**	**Count**	**%**	**Bonferroni P-value**
INTERPRO	PDZ domain	5	8.77	7.5×10^{-6}
UNIPROT_FEATURES	Fibronectin type III domain	4	7.02	3.2×10^{-5}
KEGG_PATHWAY	Natural killer cell mediated cytotoxicity	4	7.14	1.8×10^{-4}
GOTERM_BP_DIRECT	NK cell activation	4	7.14	3.4×10^{-4}
KEGG_PATHWAY	Fc gamma receptor-mediated phagocytosis	3	6.82	1.3×10^{-3}
GOTERM_CC_DIRECT	Postsynaptic density	4	7.02	1.2×10^{-3}
GOTERM_MF_DIRECT	Cell adhesion molecule binding	3	6.82	1.6×10^{-3}
GOTERM_BP_DIRECT	Positive regulation of TNF production	4	7.14	1.7×10^{-3}
GOTERM_CC_DIRECT	Membrane raft	3	6.82	2.7×10^{-3}

G More Experimental Results in Discussion

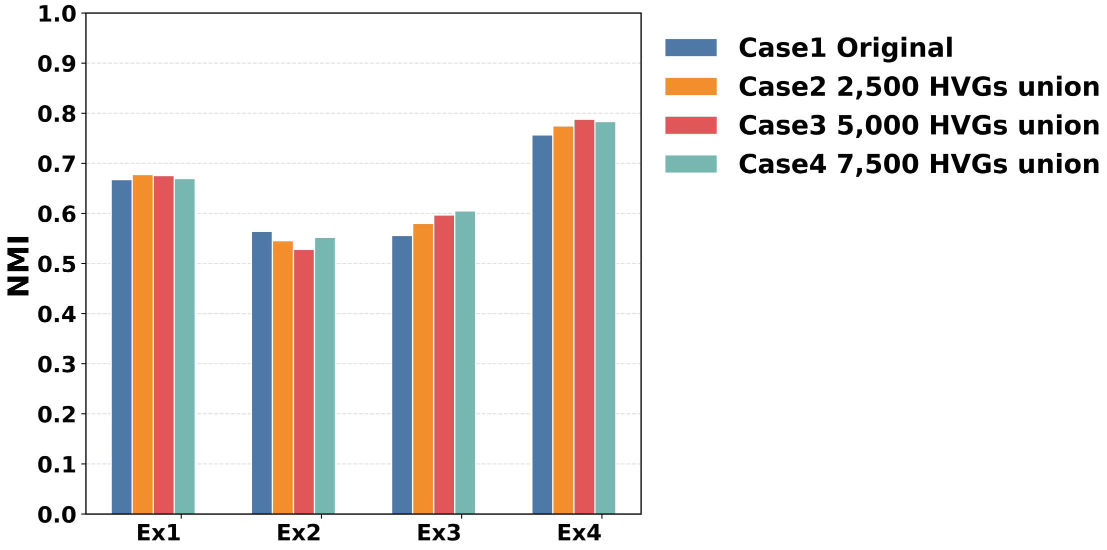

Figure A4: Clustering performance of GuidedCoC under different feature selection strategies across four datasets. Case 1: 2500 HVGs selected only from the source scRNA-seq data. Cases 2–4: Feature sets constructed as the union of HVGs from both source and target data, totaling 5000, 10,000, and 15,000 genes, respectively. Each set comprises an equal number of HVGs from both domains.

Table A3: Quantitative comparison of GuidedCoC performance using original versus feature-reordered target data $A^{(t)}$ across four real-world examples.

**Methods**	**Example 1**		**Example 2**		**Example 3**		**Example 4**	
	NMI	ARI	NMI	ARI	NMI	ARI	NMI	ARI
Original $A^{(t)}$	0.6676	0.5399	0.5640	0.4089	0.5558	0.4539	0.7570	0.8224
Reordered $A^{(t)}$	0.6551	0.5158	0.5511	0.3917	0.5452	0.3786	0.7316	0.7909

Table A4: Label transfer accuracy on Example 1 across two settings. **Bold** indicate best results.

	Setting 1		Setting 2	
	F1 (weighted)	F1 (macro)	F1 (weighted)	F1 (macro)
GuidedCoC	**0.3751**	**0.4167**	0.7332	**0.4627**
scNCL[Yan et al., 2023]	0.2304	0.2093	0.5451	0.2610
scJoint[Lin et al., 2022]	0.3180	0.2803	**0.7389**	0.3484

References

J. Chiou, C. Zeng, Z. Cheng, J. Han, M. Schlichting, M. Miller, V. A. Traag, Y. Wang, C. Dorrell, et al. Single-cell multiomic analysis of human pancreatic islets reveals novel type 2 diabetes genes. *Nature Genetics*, 55(1):136–150, 2023.

Y. Lin, T.-Y. Wu, S. Wan, J. Yang, W. Wong, and Y. Wang. scjoint integrates atlas-scale single-cell rna-seq and atac-seq data with transfer learning. *Nature Biotechnology*, 40(5):703–710, 2022. doi: 10.1038/s41587-022-01284-4.

X. Yan, R. Zheng, J. Chen, and M. Li. scncl: transferring labels from scrna-seq to scatac-seq data with neighborhood contrastive regularization. *Bioinformatics*, 39(8):btad505, 2023. doi: 10.1093/bioinformatics/btad505.
