# Supplementary figures and images for: Guided co-clustering transfer across unpaired and paired single-cell multi-omics data
Source: Bioinformatics. 2025 Dec 1;41(12):btaf639. doi: 10.1093/bioinformatics/btaf639 (PMC12696646; doi:10.1093/bioinformatics/btaf639)

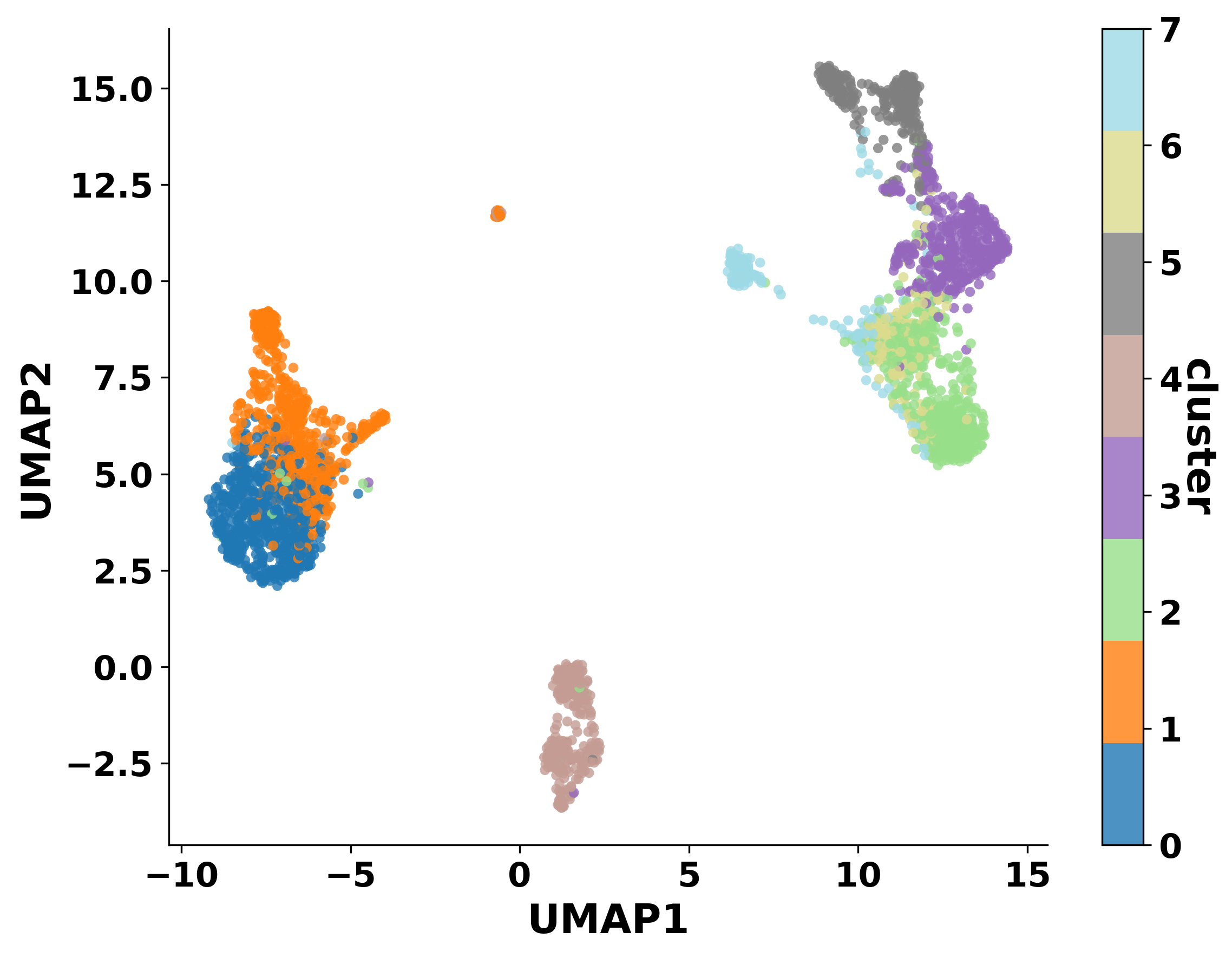

Supplement: btaf639_Supplementary_Data [file btaf639_supplementary_data.zip › Ground_truth_ex1.png]

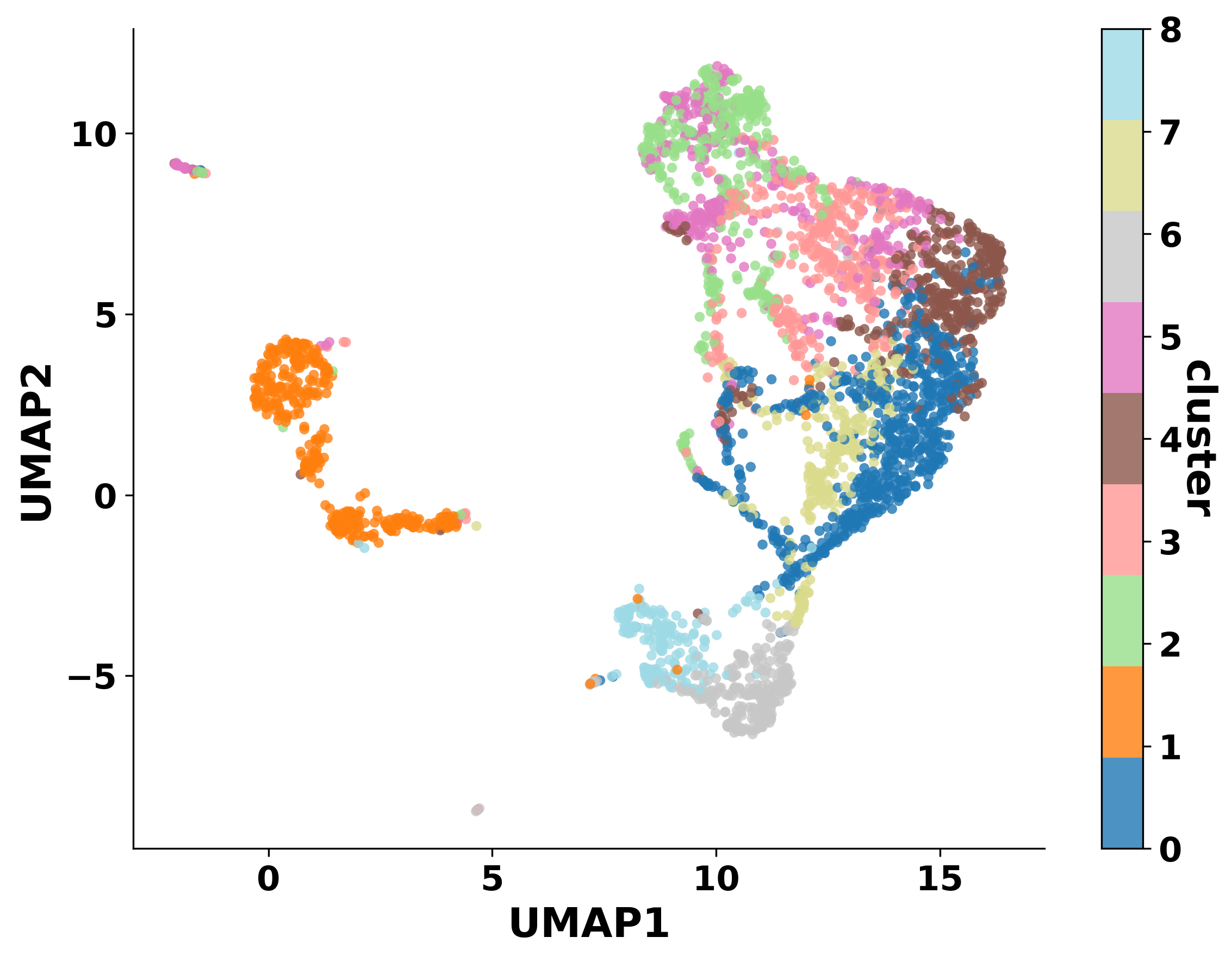

Supplement: btaf639_Supplementary_Data [file btaf639_supplementary_data.zip › Ground_truth_ex2.png]

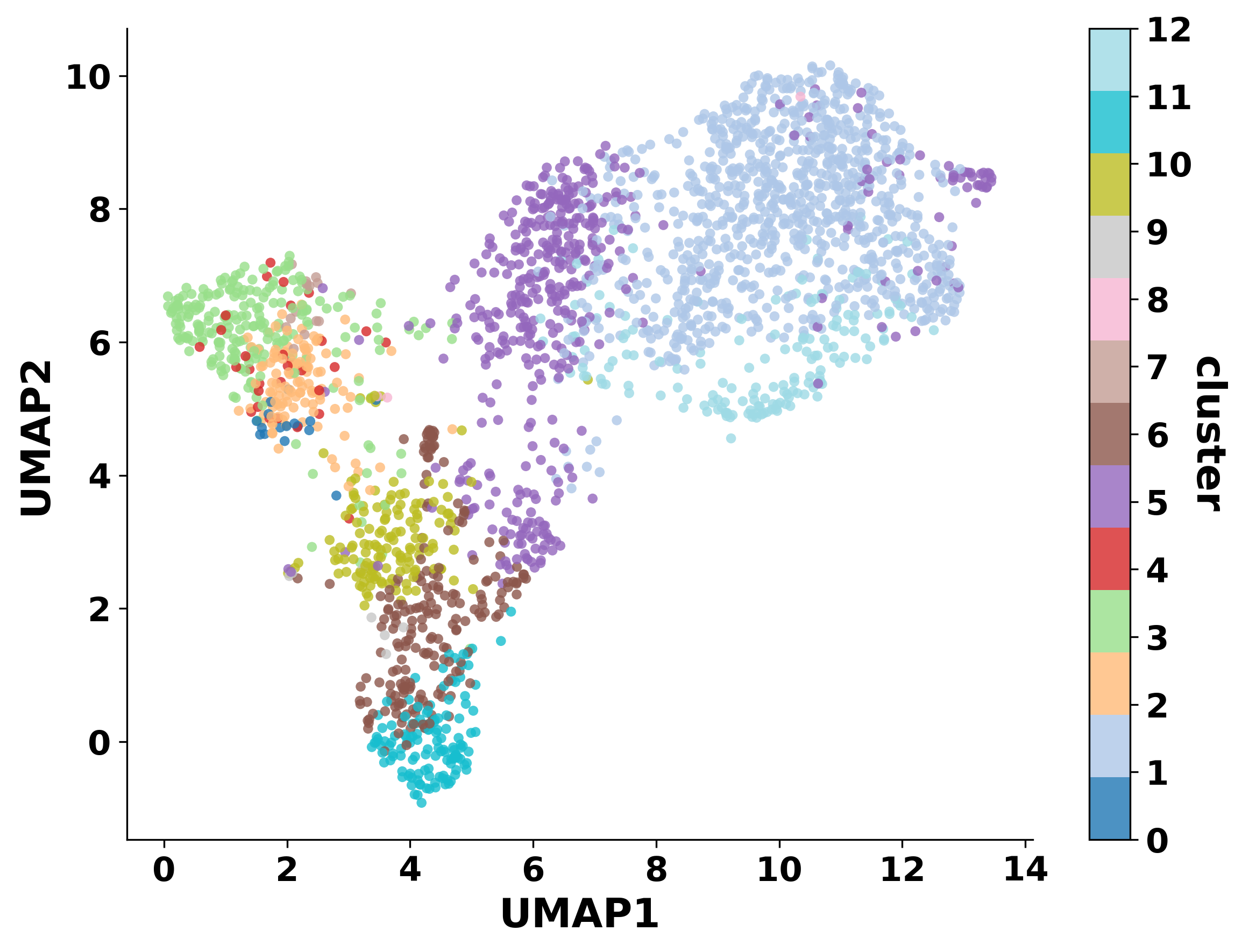

Supplement: btaf639_Supplementary_Data [file btaf639_supplementary_data.zip › Ground_truth_ex3.png]

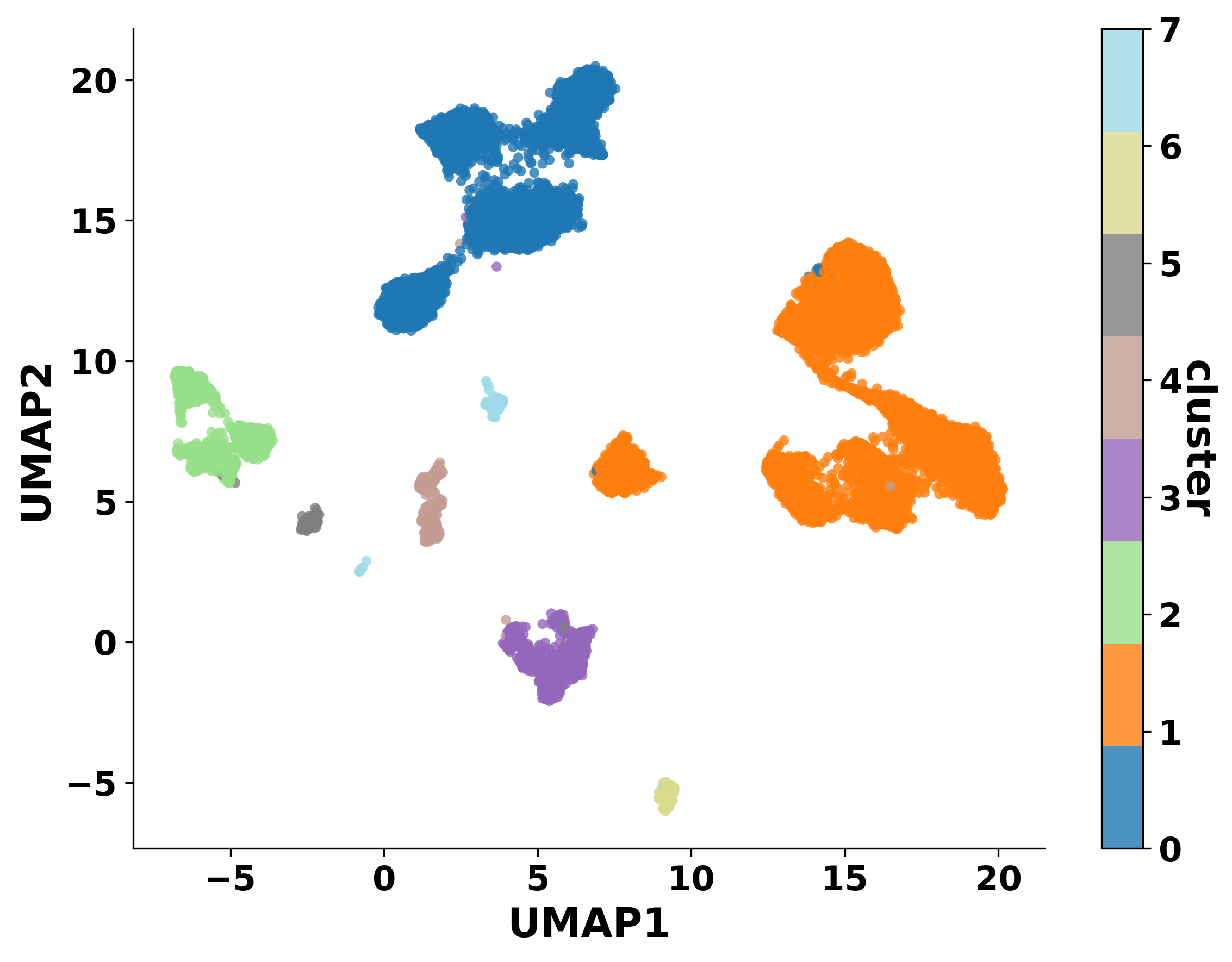

Supplement: btaf639_Supplementary_Data [file btaf639_supplementary_data.zip › Ground_truth_ex4.png]

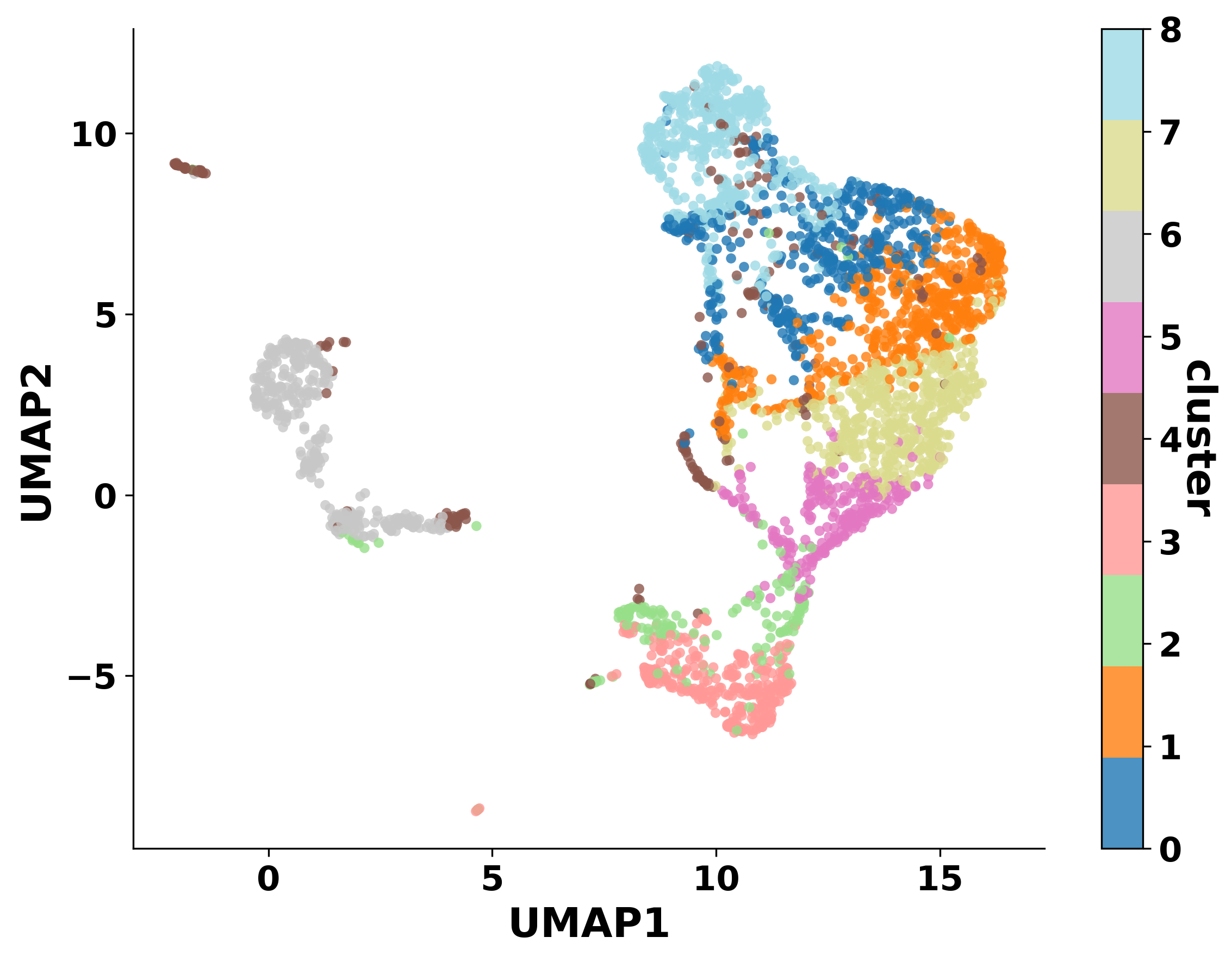

Supplement: btaf639_Supplementary_Data [file btaf639_supplementary_data.zip › GuidedCoC_ex2.png]

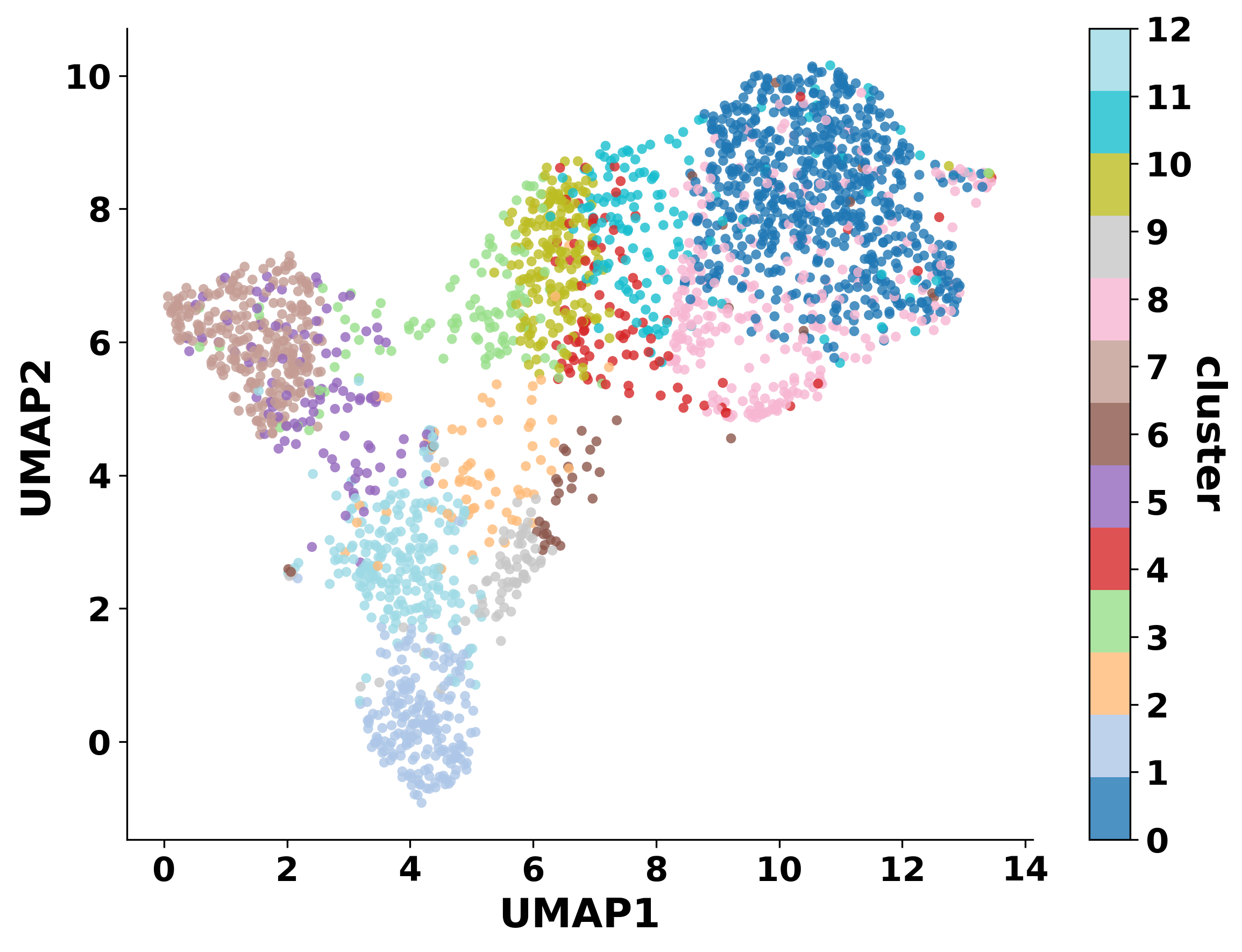

Supplement: btaf639_Supplementary_Data [file btaf639_supplementary_data.zip › GuidedCoC_ex3.png]

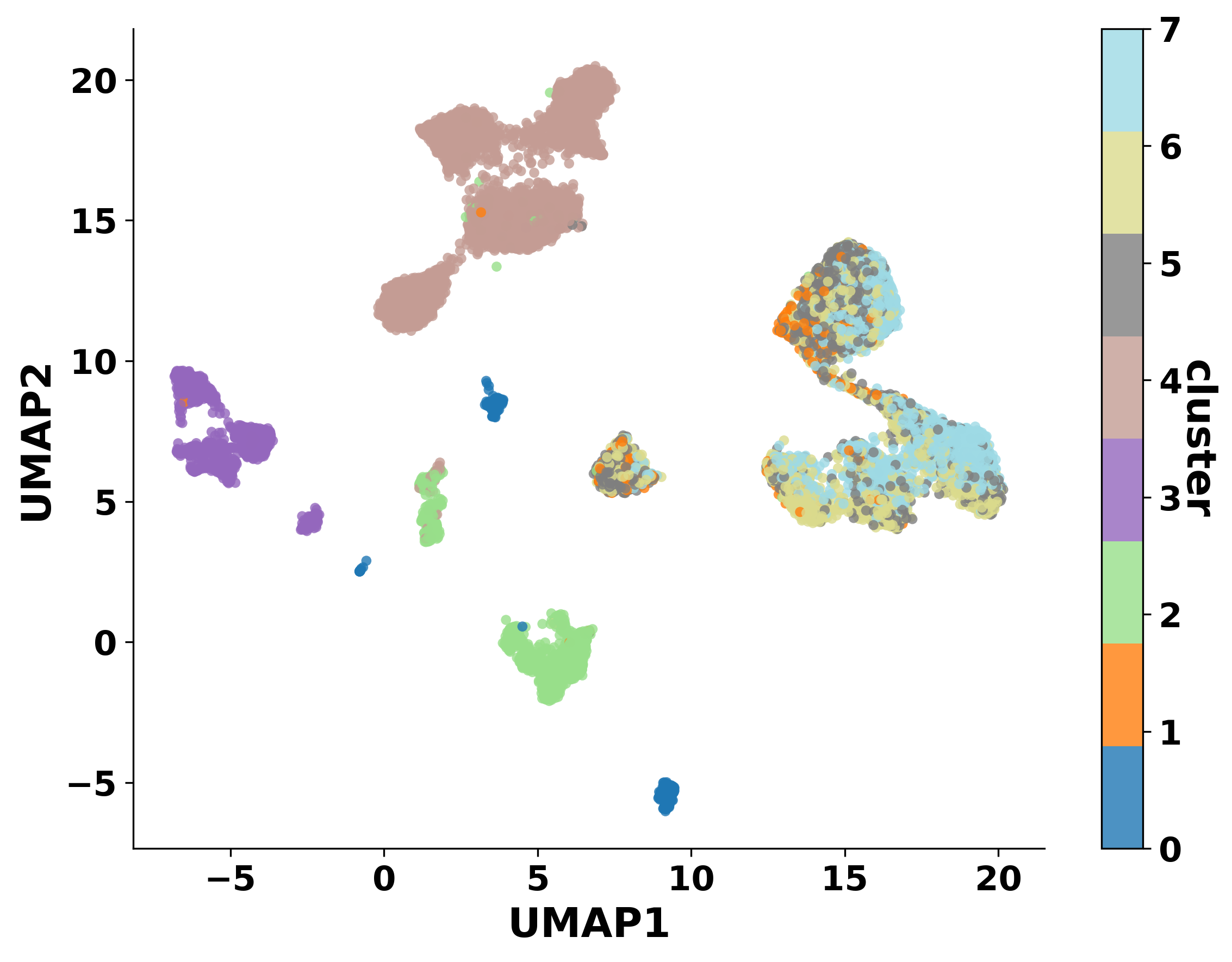

Supplement: btaf639_Supplementary_Data [file btaf639_supplementary_data.zip › GuidedCoC_ex4.png]

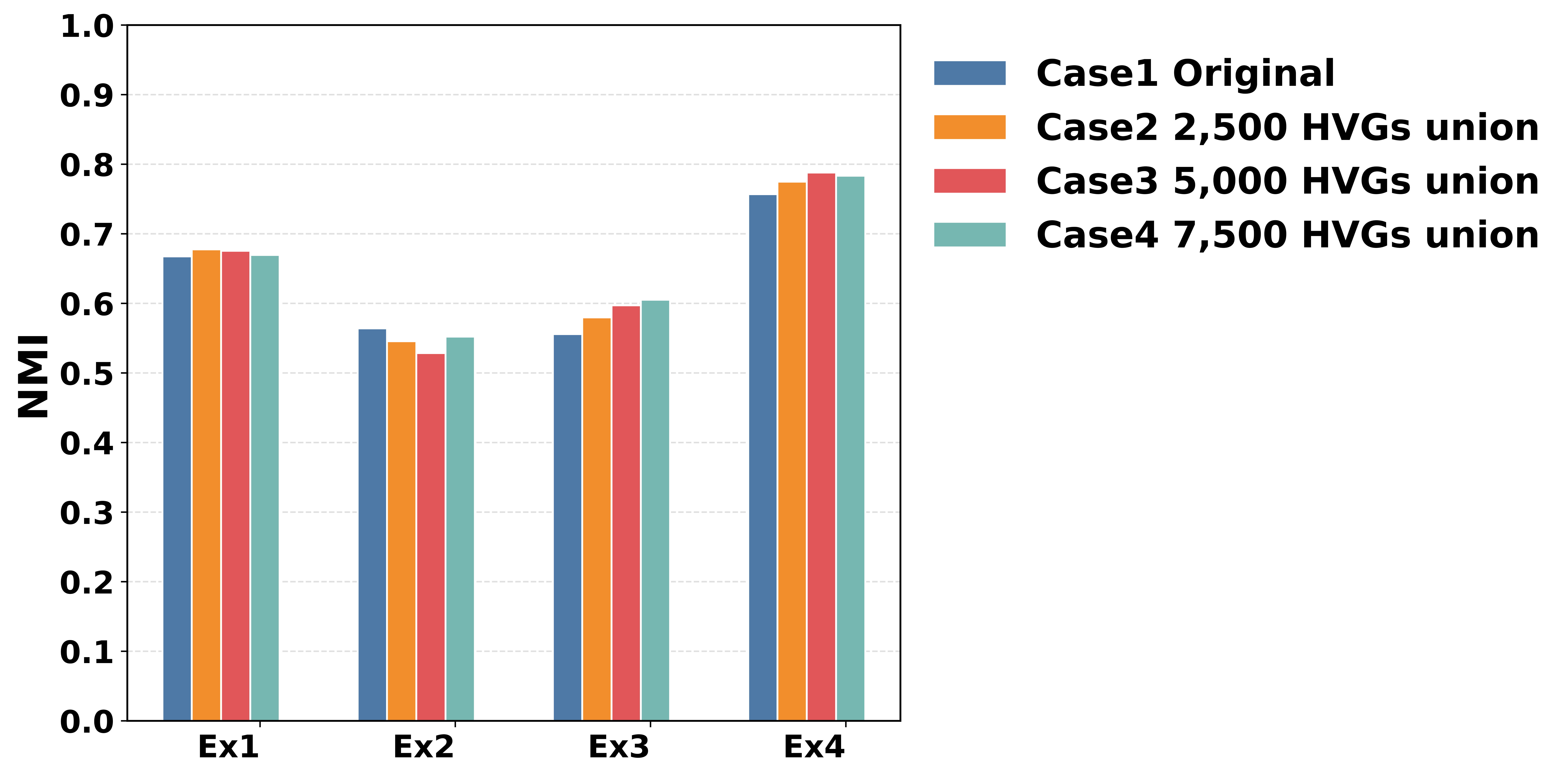

Supplement: btaf639_Supplementary_Data [file btaf639_supplementary_data.zip › HVG_Selection.png]

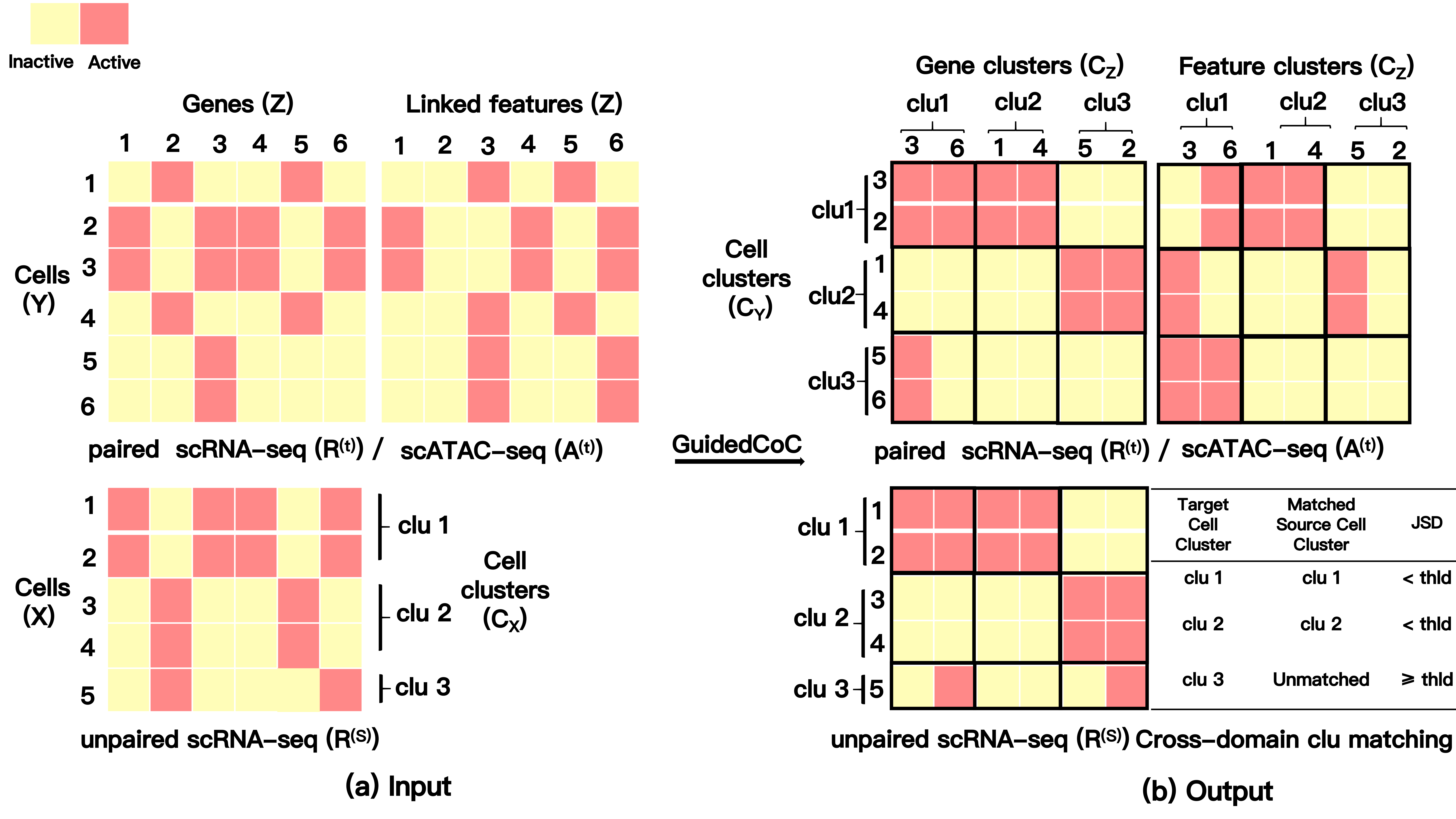

Supplement: btaf639_Supplementary_Data [file btaf639_supplementary_data.zip › overview.png]

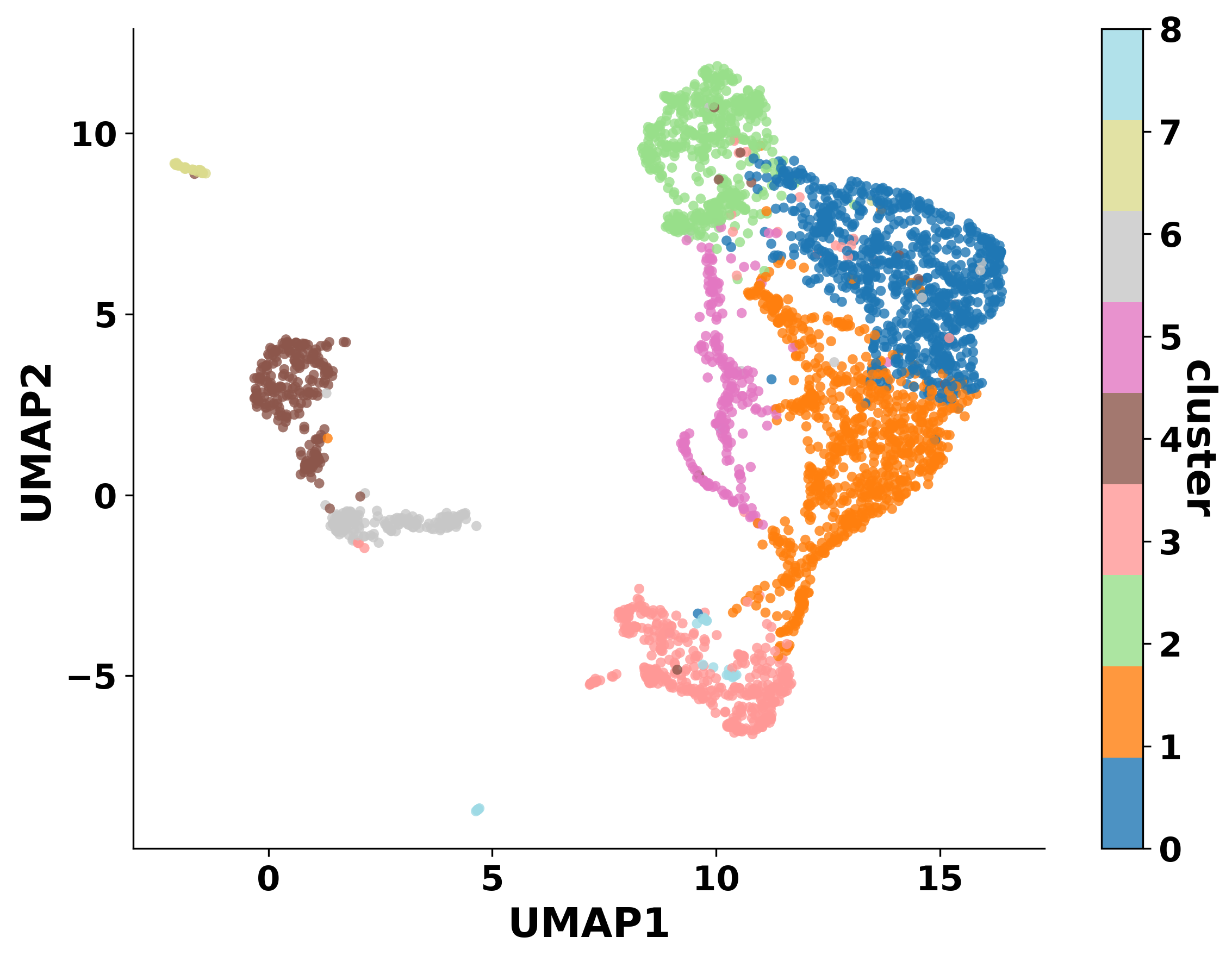

Supplement: btaf639_Supplementary_Data [file btaf639_supplementary_data.zip › Seurat5_ex2.png]

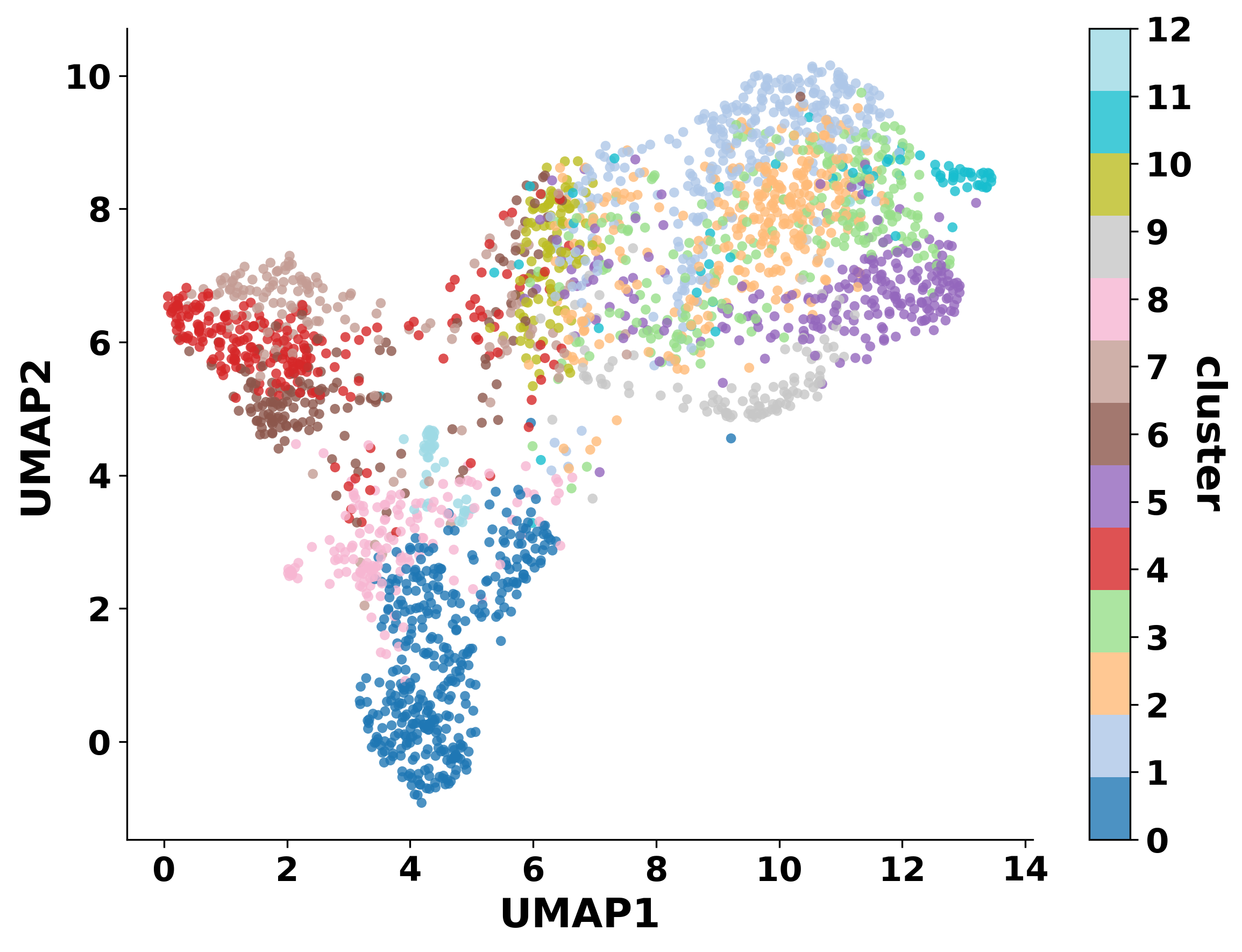

Supplement: btaf639_Supplementary_Data [file btaf639_supplementary_data.zip › Seurat5_ex3.png]

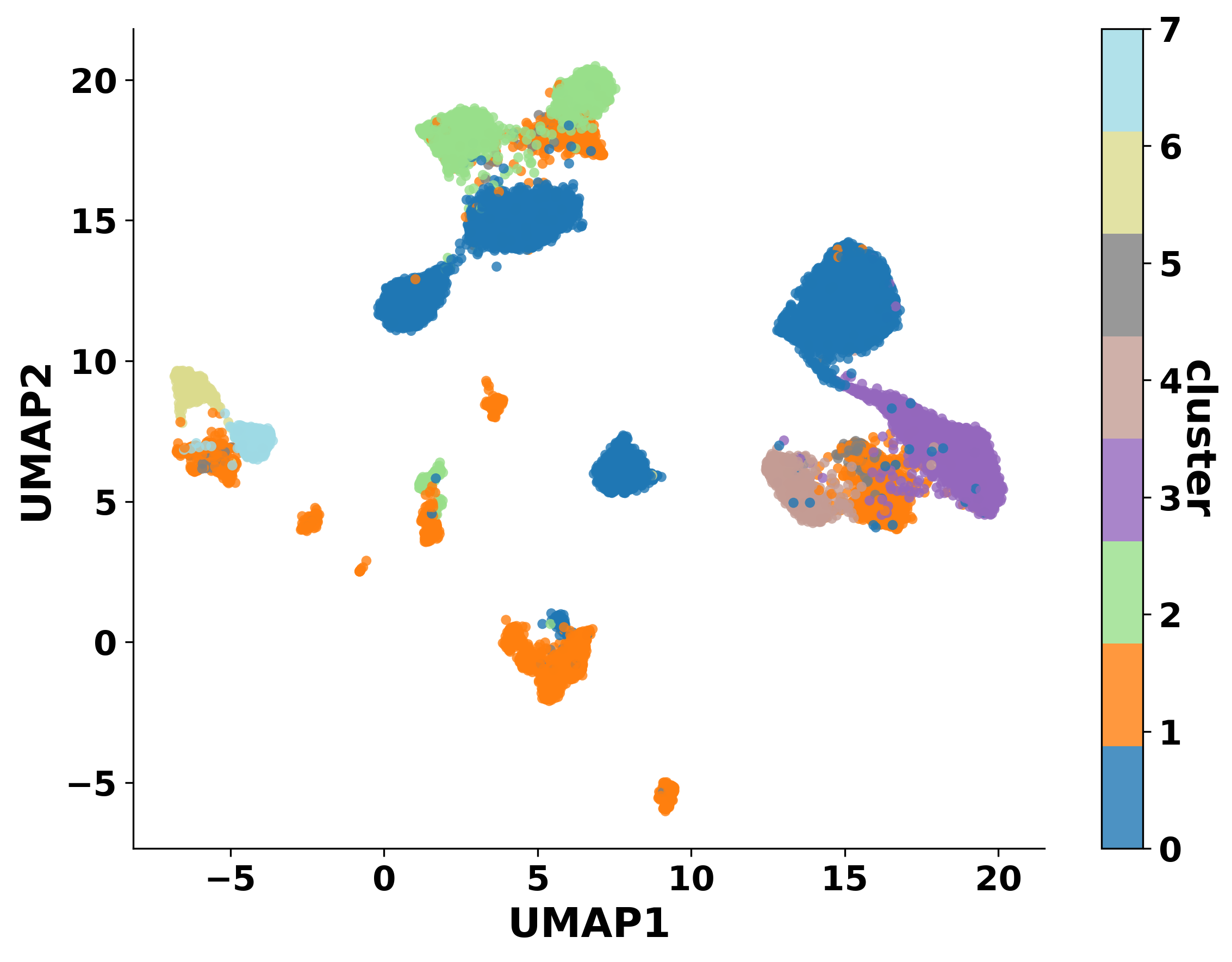

Supplement: btaf639_Supplementary_Data [file btaf639_supplementary_data.zip › Seurat5_ex4.png]
